# Comparative Phytochemical Studies on the Aerial Parts of *Teucrium davaeanum* Coss. and *Teucrium zanonii* Pamp.

**DOI:** 10.3390/molecules31122196

**Published:** 2026-06-22

**Authors:** Randa Aldaba, Azmi Hanoğlu, Duygu Yiğit Hanoğlu, Kemal Hüsnü Can Başer, Mehmet Öztürk, Ahmet Ceyhan Gören, Simon Jurt, İhsan Çalış

**Affiliations:** 1 Department of Pharmacognosy, Faculty of Pharmacy, Near East University, 99010 Lefkoşa, Turkish Republic of Northern Cyprusazmi.hanoglu@neu.edu.tr (A.H.);; 2 Department of Pharmaceutical Botany, Faculty of Pharmacy, Near East University, 99010 Lefkoşa, Turkish Republic of Northern Cyprus; 3 Department of Chemistry, Faculty of Science, Mugla Sitki Kocman University, 48121 Muğla, Türkiye; 4 Faculty of Chemistry and Chemical Technology, Al-Farabi Kazakh National University, Almaty-Kazakhstan 71, Almaty 050040, Kazakhstan; 5 Department of Chemistry, Faculty of Sciences, Gebze Technical University, 41400 Gebze, Kocaeli, Türkiye; 6 Doruk Analitik, Troyasil HPLC Column Technologies, 34000 Istanbul, Ümraniye, Türkiye; 7 Department of Chemistry, University of Zurich, Winterthurerstrasse 190, CH-8057 Zurich, Switzerland

**Keywords:** Lamiaceae, *Teucrium davaeanum*, *Teucrium zanonii*, iridoids, phenylethanoids, flavonoids, saponins, antidiabetic activity

## Abstract

Phytochemical studies performed on the aerial parts of *Teucrium davaeanum* Coss. resulted in the isolation of an iridoid diglycoside, teucardoside; two phenylethanoid triglycosides, poliumoside and 3-*O*-methyl-poliumoside; a flavon C-diglycoside, vicenin-2 (apigenin-6,8-di-C-glycoside); and a newly described bisdesmosidic oleanane-type triterpene saponin, davaeanoside. Structure elucidations of all isolated metabolites are based on extensive spectroscopic analysis and chemical derivatizations. The extract and isolated compounds (**1**–**5**) were tested for α-amylase and α-glucosidase inhibitory activity. IC50 values were measured for all extracts and compounds and compared against acarbose. Results revealed weak or moderate α-amylase and α-glucosidase inhibitory activity at the tested concentrations of the isolated compounds, especially compound **5**. However, these findings do not exclude antidiabetic activity mediated by other mechanisms such as modulation of insulin signaling, enhancement of glucose uptake, or antioxidant effects. Further studies are warranted to explore these potential pathways. In addition, the essential oils of *T. davaeanum* and *T. zanonii* were obtained by hydrodistillation and simultaneously analyzed by GC-FID and GC/MS. The major compounds of *T. davaeanum* essential oil were germacrene D (31.4%) and bicyclogermacrene (15.9%); the main compounds of *T. zanonii* were β-pinene (19.5%), α-muurolene (13.4%), oxo-7,8-dihydro-β-ionol (9.2%), and α-pinene (6.9%).

## 1. Introduction

The genus *Teucrium*, which belongs to the family Lamiaceae, comprises more than 300 species distributed globally, with a particular concentration in the Mediterranean region [1]. The genus Teucrium is mainly distributed in the Libyan geography in three separate sections as Chamaedrys (Mill.) Schreb., Teu-crium L. and Polium (Mill.) Schreb, with 11 species, five of which are endemic [2]. *Teucrium* species are recognized as rich sources of volatile oils, neo-clerodane diterpenoids, furanoid diterpenoids, and flavonoids. The genus is considered as one of the most abundant natural reservoirs of neo-clerodane diterpenoids, which serve as chemotaxonomic markers. Previous phytochemical investigations have revealed the presence of monoterpenes, sesquiterpenes, diterpenes, sterols, saponins, iridoids, flavonoids, polyphenolic compounds, alkaloids, and essential oils [1,3]. The essential oil compositions of the genus *Teucrium* have been summarized previously [4].

Historically, *Teucrium* species have been employed in traditional medicine for more than two millennia. They exhibit diverse biological activities, including diuretic, diaphoretic, antiseptic, antipyretic, antispasmodic, antiulcer, antirheumatic, antibacterial, antioxidant, hypoglycemic, and antifeedant properties. In folk medicine, *Teucrium* extracts are used to treat stomach and intestinal disorders, rheumatism, hemorrhoids, renal inflammation, and asthma. Infusions of leaves and flowers have also been used as substitutes for hops in beer flavoring [3,5,6].

Among the Libyan species, *Teucrium davaeanum* Coss. and *Teucrium zanonii* Pamp.—locally known as “Jaida”—have attracted particular interest. Previous phytochemical studies of *T. zanonii* reported the isolation of flavonoids such as cirsiliol, luteolin, chrysoeriol, and xanthomicrol from ethyl acetate extracts and apigenin-6,8-di-O-glucoside and luteolin-7-*O*-rutinoside from butanol extracts. Subsequent studies demonstrated that aqueous extracts of *T. zanonii* exhibited strong insecticidal activity against the olive bark beetle (*Phloeotribus oleae*). Furthermore, volatile oil analysis revealed 74 compounds, with germacrene-D as the major constituent, while ethyl acetate and butanol extracts showed notable antioxidant activity [3].

In contrast, *T. davaeanum* has been investigated for its promising biological activities. One of these studies demonstrated that the extracts of some Libyan plants exhibited antimicrobial properties, with three species belonging to the Labiate family, including *T. davaeanum*. This study showed that the solvent extracts (ethyl acetate, chloroform, butanol, and acetone) and volatile oils had antimicrobial activity against Gram-positive bacteria (*Staphylococcus aureus*, *Mycobacterium phlei*, and *Bacillus subtilis*), Gram-negative bacteria (*Escherichia coli*), and fungi (*Candida albicans*) [7]. An additional study reported that alcoholic extracts of *T. davaeanum* significantly reduced blood glucose levels in diabetic mice by approximately 60% after two weeks of treatment [8]. Moreover, the ethyl acetate extracts of the aerial parts of *T. davaeanum* were found to have the strongest in vitro antimicrobial activity against Gram-positive (*Staphylococcus aureus*) and Gram-negative (*Escherichia coli* and *Pseudomonas aeruginosa*) bacteria, followed by volatile oils and cold aqueous extracts [9].

Despite these findings, comprehensive phytochemical investigations of *T. davaeanum* and *T. zanonii* remain limited. Therefore, the present study aims to conduct a comparative analysis of the phytochemical compositions of these two endemic Libyan species.

## 2. Results and Discussion

**Figure 1:**
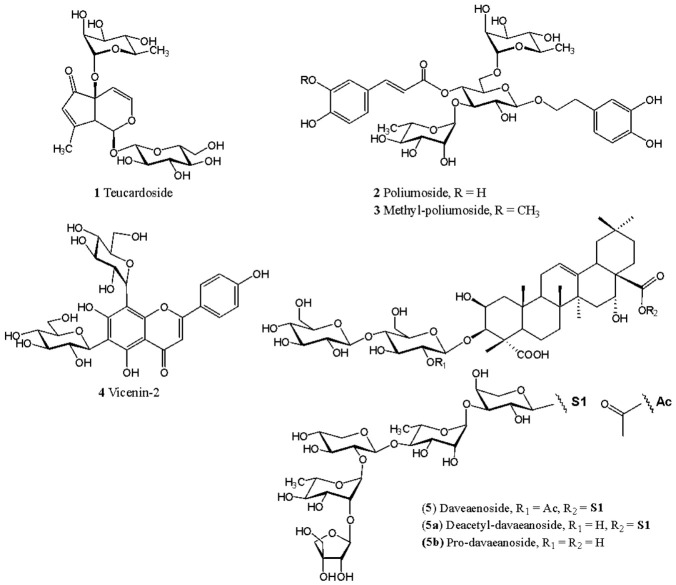
The structures of compounds **1**–**5**.

The studies were performed on the aerial parts of *Teucrium davaeanum*, resulting in the isolation of five compounds: an iridoid diglycoside, teucardoside (**1**); two phenylethanoid triglycosides, poliumoside (**2**) and methyl-poliumoside (**3**); a flavon C-diglycoside, vicenin-2 (**4**); and an oleanane-type bisdesmosidic ester saponin, davaeanoside (**5**) (Figure 1). The same compounds in the aerial parts of the *T. zanonii* were also determined with the help of chromatographic techniques (TLC and HPLC).

Compound (**1**) was obtained as a colorless, amorphous substance. HR-MS of **1** exhibited a sodium adduct ion peak at *m*/*z* 513.1578 [M + Na]^+^ compatible with the molecular formula C_21_H_30_O_13_Na^+^ (calcd. 513.1584). Its structure was deduced from ^1^H-NMR and ^l3^C-NMR as well as 2D-NMR (COSY, HSQC, HMBC, and NOESY).

The ^1^H and ^13^C-NMR data (Table 1) were indicative of an iridoid diglycoside. The ^13^C-NMR spectrum of **1** displayed twenty-one carbon resonances arising from two sugar units and a cyclopentan-pyran moiety with nine carbon atoms. The signals attributed to sugar units supported the presence of β-D-glucopyranosyl and α-*L*-rhamnopyranosyl units. Further signals were assigned to the cyclopentane-pyrane moiety at δ 6.45 (d, *J* = 6.3 Hz, H-3), 4.99 (dd, *J* = 6.3 and 1.0 Hz, H-4), 5.97 (dq, *J* = 3.3 and 1.0 Hz, H-7), 5.84 (d, *J* = 2.5 Hz, H-1), 3.54 (m, H-9), and 2.27 (3H, d, J = 1.0 Hz, H_3_-10), indicating a non-conjugated iridoid enol-ether system and a conjugated enone system in a cyclopentane ring as observed for allobetonicoside [10]. The multiplicities of the signals of H-3 and H-4 support C-5 to be substituted.

**Figure 2:**
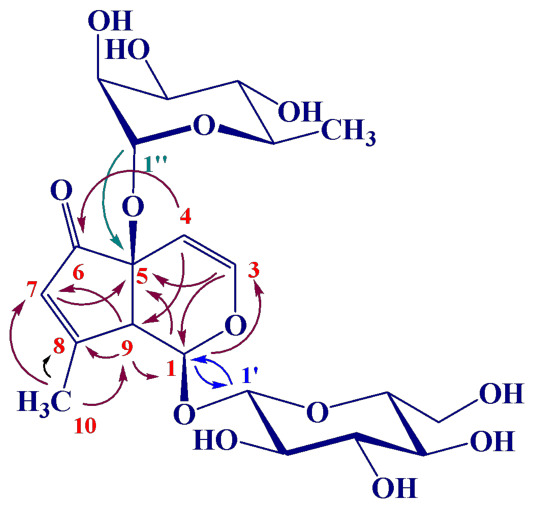
The significant ^13^C, ^1^H-long-range correlations observed in the HMBC of **1**.

This significant coupling pattern suggests a 7-ene-6-one structure, and the experimental data are also in good agreement with the reported data for teucardoside, and allobetonicoside with a similar iridoid skeleton [10,11]. The ^1^H-NMR signals of the anomeric protons at δ 4.60 (d, *J* = 7.8 Hz, H-1′) and 5.45 (d, *J* = 1.9 Hz, H-1″) as well as other protons in the same spin systems together with coupling strongly supported the presence of β-D-glucopyranose and α-L-rhamnopyranose, respectively (Table 1). The glycosidation pattern of **1** was also confirmed by the HMBC experiment. Based on the HMBC correlations, the sites of glycosylation were observed between C-1 and C-5 of the cyclopentane-pyran moiety and the anomeric protons of the β-glucose and α-rhamnose units, respectively (Figure 2). These observations show good accordance with those of teucardoside, reported first from *Teucrium polium* [11].

Compound **2** was isolated as amorphous powder. The negative-ion HRESIMS of compound **2** exhibited a pseudomolecular ion [M–H]^−^ at *m*/*z* 769.2550 compatible with the molecular formula C_35_H_45_O_19_, (calcd. for C_35_H_45_O_19_ 769.2550). The ^13^C NMR spectrum was in good agreement with the observation of two methyl, three methylene, 23 methine, and seven quaternary carbon resonances in its structure (Table 2). The ^1^H NMR spectrum of **2** exhibited the characteristic signals belonging to (*E*) caffeic acid and 3,4-dihydroxyphenylethanol moieties: protons of aromatic rings (two ABX systems), two *trans*-olefinic protons (AB system, *J*_AB_ 15.9 Hz), β-methylene at δ 2.80 (2H, m), and two nonequivalent protons at δ 3.99 and ~3.70 (each 1H, m) of the side-chain of the aglycon moiety. Additionally, three anomeric proton resonances appeared at δ 5.19, 4.63 (both d, *J* = 1.7 Hz, H-1″ and H-1‴ of two α-rhamnose) and 4.38 (d, *J* = 7.9 Hz, H-1′ of β-glucose), indicating its triglycosidic structure. Moreover, the two secondary methyl signals at δ 1.08 and 1.20 (both d, *J* = 6.2 Hz) supported the presence of two rhamnose units in **2**. The ^1^H and ^13^C-NMR data of **2** (Table 2) were in good agreement with those of poliumoside isolated from *Teucrium belion* Schreb. [12]. The carbon resonances attributed to the α-rhamnopyranosyl units were consistent for their terminal positions. Moreover, the carbon resonances at δ 81.77 and 67.67 assigned to C-3′ and C-6′ of the core sugar β-glucopyranose were observed to be shifted to downfield due to glycosylation, supporting the proposed structure. The site of esterification on the core sugar glucose was also supported by the proton signal observed at δ 5.00 (t, *J* = 9.2 Hz, H-4′).

All these observations strongly supported the presence of a structure, β-(3,4-dihydroxyphenyl)-ethyl-*O*-α-L-rhamnopyranosyl-(1 → 3)-*O*-α-L-rhamnopyranosyl-(1 → 6)-4-*O*-*E*-caffeoyl-β-D-glucopyranoside, poliumoside. Poliumoside has previously been reported from other *Teucrium* species, *T. polium* and *T. belion* [12].

Compound **3** was isolated from fraction F. The negative-ion ESI-MS spectrum displayed a pseudomolecular ion peak at *m*/*z* 783.2706 [M–H]^−^, consistent with the molecular formula C_36_H_47_O_19_^−^ (calcd. for C_36_H_47_O_19_, 783.2711). The ^1^H- and ^13^C-NMR spectra were very similar to those of poliumoside (Table 2). A singlet at δ_H_ 3.89 (3H), together with the corresponding carbon signal at δ_C_ 56.59, indicated the presence of an additional methoxy group.

Comparing the recorded NMR data with that of poliumoside (**2**), the data clearly shows that the most important difference between compound **3** and poliumoside (**2**) is likely a phenylpropanoid acid moiety linked by an ester bond. Examining the ^1^H- and ^13^C-NMR data for this acyl unit shows consistency with a feruloyl group as expected. Based on these observations and comparison with the literature data, the compound is understood to be [13], β-(3,4-dihydroxyphenyl)-ethyl-*O*-α-L-rhamnopyranosyl-(1 → 3)-*O*-α-L-rhamnopyranosyl-(1 → 6)-4-*O*-E-feruloyl-β-D-glucopyranoside, first reported from *Leucas indica* Linn. (Lamiaceae) [13].

Compound **4** (Figure 2) was obtained as a pale-yellow amorphous compound. Positive-ion ESI-MS exhibited a pseudomolecular ion peak at *m*/*z* 595.1652 consistent with the molecular formula C_27_H_31_O_15_^+^ (calcd. 595.1663 for C_27_H_31_O_15_^+^). The UV spectrum showed λ_max_ at 220, 270, and 336 nm.

The ^1^H-NMR spectrum of compound **4** exhibited signals arising from two anomeric protons at δ 4.77 and 4.72 (both d, *J* = ~10.0 Hz). Additionally, two AA′BB′ signals at δ 7.99 and 6.86 (both 2H, d, *J*_AB_ = 8.6 Hz) and two singlets at δ 6.78 and 13.67 were observed. The ^13^C-NMR spectrum displayed 15 carbon resonances attributable to a flavone skeleton and two sets of six carbon signals corresponding to two hexose units (Table 3). The chemical shifts of the carbon resonances due to two hexose units and 15 carbon resonances assigned to a flavone were consistent for a flavone di-C-glycosidic structure. The proton resonances assigned to the flavone skeleton were consistent for the sites of the glycosylation at C-6/C-8 positions of the flavone moiety. The carbon resonances attributed to the hexose units were indicative of the presence of two β-D-glucopyranosyl units (Table 3). The chemical shifts of the anomeric carbons of the glucose units at δ 73–75 were further evidence of a flavone di-C-glycosidic structure.

The NMR data were consistent with those reported for 6,8-di-C-glucosylapigenin, previously isolated from *Allophyllus edulis* (A.St.-Hil. et al.) Hieron. ex Niederl. var. *edulis* and var. *gracilis* Radlk. (Sapindaceae) [14] and later reported from *Salvia officinalis* L. [15]. This compound, known as vicenin-2, has also been reported as an anti-inflammatory constituent of *Lychnophora ericoides* Mart. leaves [16].

Compound **5** was isolated as an amorphous colorless powder. Negative-ion ESI MS exhibited a pseudomolecular ion peak *m*/*z* 1571.6737 [M-H]^−^ (calcd. 1571.6753 for C_71_H_111_O_38_). The ^1^H-NMR spectrum displayed signals due to seven anomeric protons at δ 5.57 (d, *J* = 4.2 Hz, H-1‴), 5.26 (d, *J* = 1.5 Hz, H-1′′′′′′), 5.12 (d, *J* = 2.6 Hz, H-1′′′′′′′), 5.10 (d, *J* = 1.5 Hz, H-1′′′′), 4.59 (d, *J* = 8.2 Hz, H-1′), 4.52 (d, *J* = 7.8 Hz, H-1′′′′′), and 4.43 d (d, *J* = 7.8 Hz, H-1″) and a triterpene moiety. The olefinic proton signal was at δ 5.37 (t, *J* = 3.3 Hz, H-12), and six tertiary methyl signals were at δ 1.37 (s, Me-27), 1.26 (s, 6H, Me-24, Me-25), 0.96 (s, Me-30), 0.88 (s, Me-29), and 0.77 (s, Me-26). A singlet signal with three-proton intensity at δ 2.15 was attributed to an acetoxymethyl function. The ^13^C-NMR spectrum revealed 71 carbon resonances, of which 30 were assigned to a triterpene moiety. The carbon resonances at δ 123.78 (CH, C-12) and 144.89 (C, C-13) together with an olefinic proton signal at 5.37 (H-12) were consistent with an oleanane-type triterpene as sapogenol (Table 4A,B). The assignments of all NMR signals were based on a series of 2D-NMR experiments such as COSY, TOCSY, HSQC, HSQC-TOCSY and HMBC. Two of the three carbonyl resonances at δ 182.74 and 177.13 were assigned to C-23 and C-28, respectively. The third one was due to an acetoxy group. Three oxygenated carbon resonances were observed at δ 71.30 (C-2), 74.72 (C-16) and 87.22 (C-3). The corresponding proton signals at δ 4.30 (m, H-2) and 4.11 (d, *J* = 4.0 Hz, H-3) belonged to the same spin system as assigned to H_2_-1 (δ 2.11 and 1.28), confirming their placement on ring A. With the help of a COSY experiment, the geminal proton (δ 4.49) of the third oxygenated carbon was also found in the same spin system with methylene protons at δ 1.70 and 1.41 (H_2_-15) (ring D). Further ^13^C−^1^H long-range correlations from the carbon resonances to the tertiary methyl groups on the triterpene skeleton helped to assign all proton and carbon resonances of the sapogenol moiety. Based on these results, the structure of the sapogenol was determined as zanhic acid, 2β,3β,16α-trihydroxy-olean-12-en-23,28-dioic acid [17,18,19]. The proton and carbon resonances arising from the sugar units were also established unambiguously using COSY, TOCSY, HSQC and HMBC experiments. Thus, based on the anomeric protons, the assignments of the protons belonging to each spin system and their corresponding carbon resonances have been definitively determined. First impressions of the results suggested that only one sugar unit was a pentafuranose (δ_C_ 112.53, δ_H_ 5.12, d, J = 2.6 Hz, anomeric carbon and proton resonance of a β-D-apiofuranosyl unit). The remaining signals were consistent with the presence of two hexose (two β-D-glucopyranosyl units; δ 4.59 and 4.43), two methyl pentose (two α-L-rhamnopyranosyl units; δ 5.26 and 5.10), and two pentose units (β-D-xylopyranosyl, δ 4.52; α-L-arabinopyranosyl, δ 5.57). Furthermore, the latter anomeric proton shifted downfield δ 5.57 of α-L-arabinopyranosyl, strongly suggesting that it was linked to the sapogenol moiety via an ester linkage. These findings for compound **5** were found to be in accordance with those of the poliusaposides isolated from *Teucrium polium* [18], especially those of poliusaposide A. HMBC experiments helped to assign interglycosidic linkages between the sugar units as well as on the sapogenol moiety. The long-range correlation from the C-3 (δ 87.22) of the sapogenol to the anomeric proton of one of two glucopyranose units at δ 4.59 (d, *J* = 8.2 Hz) indicated the site of the second glycosylation site on the sapogenol moiety. The HSQC-TOCSY experiment showed that the geminal proton of C-2′(OH) was shifted downfield due to esterification (δ 4.76 dd, *J* = 8.2 and 9.2 Hz, H-2′), with C-2(OH) showing the site of esterification of the acetyl function. An HMBC experiment indicated that the second β-D-glucopyranosyl unit was glycosylated at C-4′ of the first glucose unit, based on the long-range correlation from C-4′ (δ 79.78) to the anomeric proton of the second glucose unit (δ 4.43, H-1″) as well as the vice versa correlation from C-1″ (δ 104.62) to H-4′ (δ 3.70). The remaining signals were consistent with the terminal position of the second glucose unit (see Supplementary File).

Accordingly, the other six sugar units were assigned to the second sugar chain attached to the C-28 carboxyl group. The presence of a linear hexasaccharide moiety attached to the C-28 carboxyl function of the sapogenol was supported by long-range HMBC correlations between the sugar residues.

Based on the long-range correlations from the anomeric proton H-1′′′′ (δ 5.10) of a rhamnose unit to C-3‴ (δ 75.20) of arabinose, from H-1′′′′′ (δ 4.52) of xylose to C-4′′′′ (δ 83.82) of rhamnose, from H-1′′′′′′ (δ 5.26) of the second rhamnose unit to C-3′′′′′ (δ 84.83) of xylose, and finally from the anomeric proton of the terminal apiose unit H-1′′′′′′′ (δ 5.12) to the C-2′′′′′′ (δ 80.33) of the rhamnose, the structure of the hexasaccharide moiety was established as β-D-apiofuranosyl-(1 → 2)-α-L-rhamnopyranosyl-(1 → 3)-β-D-xylopyranosyl-(1 → 4)-α-L-rhamnopyranosyl-(1 → 3)-α-L-arabinopyranoside.

The interglycosidic linkages were further supported by reciprocal HMBC correlations from the anomeric carbons to the corresponding protons of the glycosylated positions.

**Figure 3:**
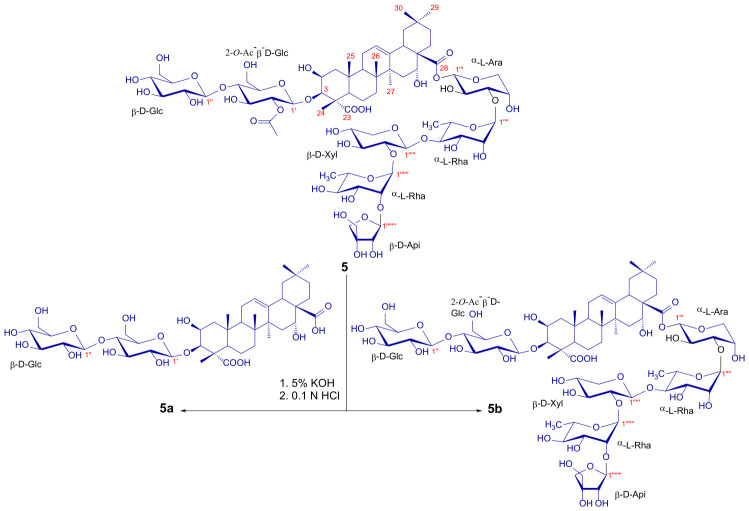
Alkaline hydrolysis of compound **5**.

To prove the structure of **5**, an aliquot of compound **5** (130 mg) was subjected to an alkaline hydrolysis yielding two compounds, **5a** (9 mg, Rf: 0.50) and **5b** (72 mg, Rf: 0.20) (Figure 3). NMR spectroscopic analysis revealed that **5a** corresponded to the prosaponin moiety of **5**, while **5b** was identified as the deacetylated derivative of **5**. These findings indicate that the alkaline hydrolysis proceeded only partially; however, the resulting deacetyl saponin and prosaponin proved valuable for elucidating the structural features of compound **5**.

Compound **5a** was obtained as an amorphous colorless powder. The low-resolution negative-ion ESI-MS spectrum exhibited a pseudomolecular ion peak at *m*/*z* 841.76 [M–H]^−^, consistent with the molecular formula C_42_H_65_O_17_ (calcd. for C_42_H_65_O_17_^−^, 841.42). The ^1^H-NMR spectrum (Table 5A) displayed signals attributable to zanhic acid as the sapogenol together with two anomeric proton signals at δ 4.43 (both d, *J* ≈ 8.0 Hz, H-1′ and H-1″), indicating the presence of two β-glucopyranosyl units glycosylated at C-3 (δC 87.10). The ^13^C-NMR spectrum (Table 5B) exhibited 42 carbon resonances consistent with zanhic acid as sapogenin and a diglycosidic sugar chain identified as β-D-glucopyranosyl-(1 → 2)-β-D-glucopyranoside. Thus, the structure of **5a** was identified as 3-*O*-[β-D-glucopyranosyl-(1 → 4)-β-D-glucopyranosyl]-zanhic acid.

Compound **5b** was obtained as an amorphous colorless powder. Negative-ion ESI exhibited a pseudomolecular ion peak at *m*/*z* 1529.6641 [M–H]^−^, consistent with a molecular formula of C_69_H_109_O_37_ (calcd. for 1529.6647 C_69_H_109_O_37_^−^). The ^1^H- and ^13^C-NMR data were in accordance with that of compound **5** except with the lack of acetyl function (Table 5A,B). Therefore, the most prominent difference was observed for the chemical shifts of the geminal protons of C-2′(OH) of the glucose unit next to the sapogenol moiety, which was observed at higher field (δ 3.30, H-2′) in comparison to that of 5 (δ 4.76, H-2′) (Table 5B). Deacylation also caused slight differences for the chemical shift values of C-2′ and C-3′ (Table 5A,B). Therefore, the structure of **5b** was identified to be deacetyl-davaeanoside.

Based on spectroscopical and chemical experiments, the structure of compound **5** was established as 3-*O*-[β-D-glucopyranosyl-(1 → 4)-2-*O*-acetyl-β-D-glucopyranosyl]-28-*O*-[β-D-apiofuranosyl-(1 → 2)-α-L-rhamnopyranosyl-(1 → 2)-β-D-xylopyranosyl-(1 → 4)-α-L-rhamnopyranosyl-(1 → 3)-α-L-arabinopyranosyl]-zanhic acid ester. The only difference between compound **5** and that of poliusaposide A isolated from *Teucrium polium* [18] was the site of glycosidation of the terminal apiose unit on the following sugar, rhamnose. Because of this small structural difference, davaeanoside was proposed as a trivial name for compound **5**.

The results presented in Table 6 demonstrate that the tested extracts have no antidiabetic potential at the tested concentrations. Moreover, the tested compounds exhibited weak inhibitory activity against both α-amylase and α-glucosidase. Specifically, most compounds exhibited IC_50_ values above 100 µg/mL for α-amylase inhibition, indicating low potency relative to the reference compound acarbose (IC_50_ = 62.92 µg/mL). However, some of the compounds, such as **5**, exhibited moderate α-glucosidase inhibition (26.52% at 50 µg/mL) with measurable percentage inhibition, suggesting selective activity. Several samples were reported as inactive (NA) or insufficient, limiting comprehensive evaluation. Overall, the compounds have weak antidiabetic potential at the tested concentrations (see Supplementary File).

The essential oils of both species (*T. davaeanum* and *T. zanonii*) were obtained by hydrodistillation and simultaneously analyzed by GC-FID and GC/MS. Only trace yields (<0.05) of the essential oils were obtained. The essential oil analysis results of *T. davaeanum* and *T. zanonii* are given in Table 7. A total of 70 compounds, representing 99.1% of the total, were identified for the essential oil of *T. davaeanum*, while 80 compounds, representing 88.2% of the total, were identified for the essential oil of *T. zanonii.* The major compounds of *T. davaeanum* oil were germacrene D (31.4%) and bicyclogermacrene (15.9%), and the main compounds of *T. zanonii* were β-pinene (19.5%), α-muurolene (13.4%), oxo-7,8-dihydro-β-ionol (9.2%), and α-pinene (6.9%).

In a previous study done in Libya, the major compounds were determined as β-pinene (14.13%), linalyl acetate (11.10%), linalool (11.00%), and germacrene D (8.81%) in the essential oil composition of *T. zanonii* [20]. Another previous study from Libya on the essential oil composition of several *Teucrium* species including *T. davaeanum* and *T. zanonii* reported the major compounds as β-caryophyllene (23.17%), germacrene B (25.17%) and limonene (8.32%) for the latter, while the major compounds for the former were β-caryophyllene (24.28%), germacrene B (21.92%) and limonene (7.26%) [21].

## 3. Materials and Methods

### 3.1. Experiments

Isolation procedures for secondary metabolites were performed using column chromatography and gradient medium-pressure liquid chromatography (Büchi MPLC, equipped with C-601 and C-605 pump modules, C-610 pump controller, and C-605 pump manager) with a Büchi C-615 fraction collector. Silica gel (0.063–200 mμ, Merck, Darmstadt, Germany) and LiChroprep C-18 (0.063–200 mm, Merck) stationary phases were used in normal- and reverse-phase column chromatography studies, respectively. Sephadex LH-20 was also used in purification studies of triterpene glycosides with high molecular weights. For further purification studies and visual determination of purity of fractions that could not be purified by column chromatography, thin-layer chromatography was used, employing Silica Gel 60 F254, Merck, alumina plates. NMR measurements of the isolated secondary metabolites were performed on Bruker DRX 600 and Bruker Neo Ascend 500 MHz spectrometers operating at 600 and 500 MHz for ^1^H NMR in DMSO-d6, and at 150 and 125 MHz for ^13^C, using the XWIN NMR 3.5 software package for data acquisition and processing. High-resolution mass spectrometry (HRMS) measurements were performed using an Orbitrap Exploris mass spectrometer (Thermo Fisher Scientific Inc., Waltham, MA, USA) with an ESI source in negative- and positive-ion modes, while GC-MS measurements were performed using a Thermo Scientific Trace GC 1300 system coupled to a Thermo TSQ 9610 MS-MS. Optical rotation measurements were performed on a Schmidt+Haensch Polartronic MHZ-8 polarimeter. Lyophilization was performed using the CHRIST Alpha 1–4 LD Plus.

### 3.2. Plant Material

The species *Teucrium davaeanum* was collected from the Gaminis region of Libya in April 2019, while the species *Teucrium zanonii* was collected and identified by Dr. Salem Bscher from the Al-Maqrun region during the same month in its flowering season. Herbarium specimens of these plants are kept in the Herbarium of the Botany Department, Faculty of Science, Tripoli University, under codes ULT 414 and ULT 387, respectively.

### 3.3. Chemicals

Dimethyl sulfoxide-d_6_ (DMSO-d6, 99.9% D, Sigma Aldrich, Darmstadt, Germany), methanol-D4 (99.95%, D, Cambridge isotope Lab. Inc., Andover, MA, USA), potassium hydroxide (Tekkim, 99%, Bursa, Türkiye), and hydrochloric acid (37%, Sigma Aldrich, Darmstadt Germany) were used in the experiments. All solvents were reagent grade and were obtained from Merck (Germany).

### 3.4. Extraction and Fractionation

Air-dried aerial parts of *T. davaeanum* were macerated with 80% EtOH (3.0 L) at room temperature for two days with periodic shaking and then filtered. The plant residue was washed with 80% EtOH, and the combined filtrates were concentrated under reduced pressure. The resulting extract was diluted with H_2_O (200 mL) and successively partitioned with dichloromethane (DCM, 200 mL × 3). The combined DCM layers were evaporated to afford a lipophilic extract (7.28 g, 3.14%), while the aqueous phase evaporated to dryness to yield the crude extract (28.58 g, 14.26%). The crude extract was fractionated by vacuum liquid chromatography (VLC) on a LiChroprep C18 (100 g) using a stepwise MeOH–H_2_O gradient, increasing the methanol content by 5% for every 100 mL of solvent. Twenty fractions were collected. Fractions 1–6 were collected in 100 mL each (0–20% MeOH). Fractions 7–20 were collected in 50 mL each (25–100% MeOH). According to the TLC profiles, twenty fractions were combined into nine fractions, A–I (A: frs. 1–2, 11.59 g; B: frs. 3–5, 862 mg; C: frs. 6–7, 522 mg; D: frs. 8–9, 775 mg; E: frs. 10–13, 3.707 g; F: frs. 14–15, 1.244 g; G: frs. 16–17, 1.001 g; H: fr. 18, 1.287 g; I: frs. 19–20, 694 mg).

Fr. A was rich in sucrose and therefore not further processed. Fr. B (862 mg) was subjected to silica gel column chromatography (65 g) using a DCM–MeOH–H_2_O mixture as the solvent system (70:30:3, 600 mL; followed by 75:25:4, 200 mL), affording compound **1** (teucardoside, 126 mg).

Fr. C (522 mg) was chromatographed on a silica gel column (50 g) using a DCM–MeOH–H_2_O mixture of increasing polarity (80:20:2, 450 mL; 75:25:2.5, 200 mL; and 70:30:3, 200 mL). The compounds obtained from this fraction decomposed immediately due to their very high hygroscopic properties, and structural determinations could not be carried out.

Fr. D (775 mg) was initially subjected to gel filtration chromatography on a Sephadex LH-20 (∅ 3 cm, h 28 cm) using MeOH–acetone (1:1, 500 mL) as the mobile phase. Fifty fractions were collected. Fractions 30–33 (111 mg), which were rich in **2**, were further purified by silica gel column chromatography (12 g) using DCM–MeOH–H_2_O (70:30:3, 300 mL) as the solvent system to yield compound **2** (poliumoside, 36 mg). Fractions 40–45 afforded compound **4** (vicenin-2, 50 mg).

Fr. E (3.707 g), which was rich in poliumoside (**2**), was subjected to MPLC on a LiChroprep C18 (∅ 25 mm, h 40 cm) using a MeOH–H_2_O mixture with an increasing proportion of MeOH. Sample application was carried out with 5% MeOH for the first 5 min. Elution was then continued with a linear gradient from 5% to 50% MeOH over 55 min, followed by an increase from 50% MeOH to 100% MeOH over 20 min. Fractions were collected at a flow rate of 10 mL/min. Fractions 63–68 yielded compound **3** (methyl-poliumoside, 21 mg), whereas fractions 71–84 afforded **2** (poliumoside, 1363 mg).

Fr. F (1.244 g) was run on silica gel column chromatography (75 g) using DCM–MeOH–H_2_O solvent systems (80:20:2, 250 mL; 75:25:2.5, 200 mL; 70:30:3, 600 mL; and 60:40:4, 100 mL). Compounds **2** (poliumoside, 41 mg) and **3** (methyl-poliumoside, 262 mg) were obtained as pure secondary metabolites from this column chromatography process.

According to the TLC data of Fr. H (1135 g), instead of direct normal- or reverse-phase column chromatography, this fraction was initially subjected to Sephadex LH-20 (∅ 3 cm, h 28) gel filtration chromatography. The mobile phase used was a MeOH–acetone–H_2_O (4:1:1, 500 mL) solvent system, and thirty fractions were collected during this column process. When these fractions were carefully examined, fractions 12–14 (111 mg), 15–21 (741 mg) and 22–24 (114 mg) were observed to be rich in compound **5** with varying degrees of purity. Thus, fractions 15–21 (741 mg) were further purified by silica gel column chromatography (70 g) using the DCM–MeOH–H_2_O solvent system (80:20:2, 250 mL; 70:30:3, 200 mL; 60:40:4, 200 mL; and 50:50:5, 200 mL), resulting in eighty (80) fractions containing approximately 10 mL of eluent per fraction. Among these fractions, fractions 62–65 (262 mg), 66–67 (79 mg), and 68–73 (101 mg) were determined to be rich in compound **5** with varying degrees of purity (total yield: 442 mg). Of these fractions, the purest fractions, fractions 66–67 (79 mg), were used for spectroscopic analysis.

### 3.5. Alkaline Hydrolysis of Compound **5**

Compound **5** (130 mg) was weighed into a reaction flask and hydrolyzed in 10% KOH solution at 50 °C for 30 min with magnetic stirring. After neutralizing the solution obtained from basic hydrolysis with 1 N HCl, the reaction mixture was purified by silica gel column chromatography (10 g) using DCM–MeOH–H_2_O (80:20:2) as the solvent system, affording two products (Figure 3). The compound with a higher Rf value (0.48) compared to compound **5** (Rf = 0.30) was obtained in a 9 mg yield and designated **5b**, whereas the second product, **5a**, exhibited a lower Rf value (0.19) (solvent system for the TLC: DCM–MeOH–H_2_O (61:32:7). NMR spectroscopic analysis revealed that **5b** corresponded to the prosaponin moiety of **5**, while **5a** was identified as the deacetylated derivative of **5**. Two compounds were isolated, **5a** (9 mg, Rf: 0.48) and **5b** (72 mg, Rf: 0.19). The former, **5a**, was more polar compared to compound **5** (Rf: 0.30), while **5b** was apolar.

### 3.6. Antidiabetic Activity Studies on the Extract and Compounds (**1**–**5**)

#### 3.6.1. Determination of α-Glucosidase Inhibitory Activity

The α-glucosidase inhibitory activity was assessed using a spectrophotometric method in which *p*-nitrophenyl-α-D-glucopyranoside served as the substrate, with acarbose as the positive control [22,23]. Following incubation of the enzyme with the test samples at 37 °C, the substrate was introduced, and the reaction was subsequently halted with sodium carbonate. The absorbance was then measured at 400 nm utilizing a microplate reader. Results were expressed as IC_50_ values and inhibition percentages at a concentration of 50 µg/mL.

#### 3.6.2. Determination of α-Amylase Inhibitory Activity

α-amylase inhibitory activity was measured using a modified spectrophotometric method where water-soluble starch (Sigma-Aldrich, Germany) was used as the substrate and acarbose as the positive control [22,24]. After incubating the enzyme with the inhibitor at various concentrations for 10 min at 37 °C, the substrate was added, and the reaction was stopped with HCl. Lugol’s iodine solution was used to monitor enzyme activity at 565 nm via a microplate reader. Results are expressed as µg of acarbose equivalent per mg of extract.

### 3.7. The Isolation of Essential Oils

After drying the aerial parts of the plants in the shade, they were chopped into small pieces and subjected to hydrodistillation in 1 L of distilled water for 3 h by using a modified Clevenger-type apparatus in 100 g portions. The resulting essential oils were collected from the Clevenger apparatus, dried over Na_2_SO_4_, and stored at 4 °C until analysis.

#### 3.7.1. GC/MS Analysis

The GC-MS measurements were performed on an Agilent 5975 GC-MSD Ssanta Clara, California, USA) system. An Innowax FSC column (60 m × 0.25 mm, 0.25 mm film thickness) was used with helium as the carrier gas (0.8 mL/min). The GC oven temperature was kept at 60 °C for 10 min, programmed to 220 °C at a rate of 4 °C/min, held constant at 220 °C for 10 min and then programmed to 240 °C at a rate of 1 °C/min. The split ratio was adjusted at 40:1. The injector temperature was set at 250 °C. Mass spectra were recorded at 70 eV. The mass range was adjusted from *m*/*z* 35 to 450.

#### 3.7.2. GC-FID Analysis

GC-FID measurements were performed using an Agilent 6890N GC system with an attached FID (flame ionization detector). The FID temperature was set to 300 °C. The same GC column and oven program were used to obtain the same elution sequence as with GC-MS; therefore, this information is not reported here to avoid duplication (see Section 3.7.1). The relative percentage amounts of the identified compounds were calculated separately for each substance from the FID chromatograms. Essential oil components were identified by comparing relative retention times with retention times of authentic samples or by comparing linear retention indices (LRIs) with a range of n-alkanes. For identification, computer mapping was used against commercial libraries (Wiley GC/MS Library, NIST Library) [25,26] and the in-house “Başer Essential Oil Components Library,” which was created using components of real compounds and known oils as well as the MS literature data [27].

## 4. Conclusions

The studies on the aerial parts of *Teucrium davaeanum* resulted in the isolation of five compounds: an iridoid diglycoside, teucardoside (**1**); two phenylethanoid triglycosides, poliumoside (**2**) and methyl-poliumoside (**3**); a flavon C-diglycoside, vicenin-2 (**4**); and an oleanane-type bisdesmosidic ester saponin, davaeanoside (**5**). Teucardoside (**1**) and poliumoside (**2**) have been previously reported from *Teucrium* species. Methyl-poliumoside (**3**) and the flavon C-diglycoside vicenin-2 (**4**) have been isolated for the first time from *Teucrium* species. Davaeanoside (**5**), a triterpene saponin, has not been previously described; however, it has a very similar structure to poliusaposide A isolated from *Teucrium polium* [18]. Moreover, this study is the second to describe the complex structure of a saponin, including full NMR data.

Additionally, compounds **1**–**5** detected in the aerial parts of *T. zanonii* were also identified using chromatographic techniques (TLC and HPLC).

Under the tested conditions for α-amylase and α-glucosidase inhibitory activity, the crude extract, fractions, and isolated compounds (**1**–**5**) showed no significant α-amylase and α-glucosidase inhibitory activity, indicating no evidence of antidiabetic potential via these enzyme-inhibitory mechanisms. However, consistent with reports on other *Teucrium* species, alternative pathways such as glucose uptake enhancement and modulation of insulin signaling may still underlie possible antidiabetic effects [28,29,30,31].

## Figures and Tables

**Table 1:** The ^1^H and ^13^C-NMR data of **1** (teucardoside) (δ_H_: 600 MHz; δ_C_: 150 MHz; CD_3_OD).

	1 (Teucardoside)		
C/H Atom	DEPT 135	δ_C_ ppm	δ_C_ ppm, *J* (Hz)
Agly			
1	CH	93.17	5.84 d (2.5)
3	CH	144.91	6.45 d (6.3)
4	CH	103.27	4.99 dd (6.3/1.0)
5	C	77.66	-
6	C	204.89	-
7	CH	129.37	5.97 t dq (1.5/3.3)
8	C	175.82	-
9	CH	55.52	3.54 m
10	CH_3_	18.40	2.27 t (1.2)
Glc			
1′	CH	99.64	4.60 d (7.8)
2′	CH	74.85	3.27 dd (7.8/9.0)
3′	CH	78.03	3.38 t (9.0)
4′	CH	71.74	3.28 t (9.0)
5′	CH	78.54	3.33 m
6′	CH_2_	62.91	3.92 dd (12.0/2.1), 3.67 dd (12.0/6.2)
Rham			
1″	CH	97.85	5.45 d (1.9)
2″	CH	73.03	3.84 dd (1.9/3.4)
3″	CH	72.25	3.71 dd (3.4/9.4)
4″	CH	74.16	3.36 t (9.4)
5″	CH	70.58	3.77 dq (9.4/6.4)
6″	CH_3_	18.40	1.20 d (6.4)

**Table 2:** The ^1^H and ^13^C-NMR data of **2** (poliumoside) and **3** (3-*O*-methyl-poliumoside) (**3**) (δ_H_ 500 MHz, δ_C_ 125 MHz; CD_3_OD).

C/H Atom		2 (Poliumoside)		3 (3-*O*-Methyl-Poliumoside)	
	DEPT135	δ_C_ ppm	δ_H_ ppm, *J* (Hz)	δ_C_ ppm	δ_H_ ppm, *J* (Hz)
Agly 1	C	131.53	-	131.53	-
2	CH	117.24	6.70 d (2.0)	117.24	6.70 d (2.1)
3	C	146.29	-	146.25	-
4	C	144.86	-	144.82	-
5	CH	116.65	6.69 d (8.2)	116.50	6.69 d (8.0)
6	CH	121.42	6.58 dd (8.2/2.0)	121.42	6.57 dd (8.0/2.0)
α	CH_2_	72.59	3.99 m/3.65–3.75 †	72.56	3.99 m/3.65–3.75 †
β	CH_2_	36.82	2.80 m	36.79	2.80 m
Glc 1′	CH	104.50	4.38 d (7.9)	104.48	4.38 d (7.9)
2′	CH	76.31	3.38 dd (7.9/9.2)	76.29	3.39 dd (7.9/9.0)
3′	CH	81.77	3.81 dd (9.2/9.2)	81.64	3.81 dd (9.0/9.0)
4′	CH	70.59	5.00 dd (9.2/9.0)	70.55	5.00 dd (9.0/9.0)
5′	CH	74.86	3.50–3.61 †	74.82	
6′	CH_2_	67.68	3.74 dd (11.4/2.1) 3.47 dd (11.4/5.4)	67.71	3.76 dd (11.4/2.1) 3.47 dd (11.4/5.5)
Rha_1_ 1″	CH	103.22	5.19 d (1.7)	103.14	5.20 d (1.7)
2″	CH	72.14	3.92 dd (1.7/3.4)	72.12	3.92 dd (1.7/3.2)
3″	CH	72.42	3.68 dd (3.4/9.5)	72.40	3.68 dd (3.2/9.5)
4″	CH	73.90	3.29 dd (9.5/9.5)	73.88	3.30 dd (9.5/9.5)
5″	CH	70.02	3.50–3.61	70.02	3.65–3.75
6″	CH_3_	18.16	1.08 d (6.3)	18.15	1.09 d (6.2)
Rha_2_ 1‴	CH	102.43	4.63 d (1.7)	102.41	4.63 d (1.7)
2‴	CH	72.20	3.84 dd (1.7/3.4)	72.19	3.85 dd (1.7/3.2)
3‴	CH	72.49	3.57 dd (3.4/9.5)	72.47	3.58 dd (3.2/9.5)
4‴	CH	74.08	3.34 dd (9.5/9.5)	74.06	3.35 dd (9.5/9.5)
5‴	CH	70.50	3.50–3.61 †	70.50	3.65–3.75
6‴	CH_3_	18.59	1.20 d (6.3)	18.55	1.20 d (6.2)
Acyl 1″″	C	127.79	-	127.79	-
2′′′′	CH	114.84	7.06 d (1.8)	111.49	7.20 d (2.0)
3′′′′	C	146.99	-	149.50	-
4′′′′	C	149.96	-	150.92	-
5′′′′	CH	115.36	6.78 d (8.2)	116.63	6.82 d (8.2)
6′′′′	CH	123.39	6.96 dd (8.2/1.8)	124.49	7.09 dd (8.2/2.0)
α′	CH	116.49	6.28 d (15.9)	115.28	6.36 d (15.9)
β′	CH	148.20	7.60 d (15.9)	148.07	7.67 d (15.9)
C=O	C	168.13	-	168.09	-
OCH_3_	CH_3_	-	-	56.59	3.89 s

† Signal patterns are unclear due to overlapping. Abbreviations: Agly, aglycone; Glc, glucose; Rha, rhamnose.

**Table 3:** The ^1^H and ^13^C-NMR data of **4** (vicenin-2) (δ_H_ 600 MHz, δ_C_ 150 MHz; DMSO-d_6_).

**C/H** **Atom**		**4 (Vicenin-2)**	
	**DEPT135**	**δ_C_ ppm**	**δ_H_ ppm, *J* (Hz)**
Aglycone			
2	C	164.07	-
3	CH	102.61	6.78 s
4	C	182.32	-
5	C	158.59	-
6	C	128.73	-
7	C	161.22	-
8	C	103.85	-
9	C	155.09	-
10	C	105.30	-
1′	C	121.53	-
2′/6′	CH	129.05	7.99 d (8.6)
3′/5′	CH	115.83	6.86 d (8.6)
4′	C	161.22	-
5-OH	-	-	13.67 s
Glc-(C-6/C-8)			
1″/1‴	CH	74.09/73.37	4.77 d (9.7)/4.72 d (10.0)
2″/2‴	CH	71.94/70.91	3.74–3.18 †
3″/3‴	CH	78.85/77.83	3.74–3.18 †
4″/4‴	CH	70.55/69.05	3.74–3.18 †
5″/5‴	CH	81.98/80.89	3.74–3.18 †
6″/6‴	CH_2_	61.29/59.79	3.74–3.18 †

† Signal patterns are unclear due to overlapping.

**Table 4:** (**A**). The ^1^H and ^13^C-NMR data of **5** (sapogenol moiety) (δ_H_ 600 MHz, δ_C_ 150 MHz; MeOD). (**B**). The ^1^H and ^13^C-NMR data of **5** (sugar moiety) (δH 600 MHz, δC 150 MHz; MeOD).

(**A**)					
	**5**				
**C/H** **Atom**	**DEPT** **135**	**δ_C_ ppm**	**δ_C_ ppm, *J* (Hz)**		**HMBC: Long-Range Correlations from H to C**
1	CH_2_	44.84	2.11 dd (14.2/2.0)/1.28 †		
2	CH	71.80	4.30 m		C-4, C-10
3	CH	87.22	4.11 d (4.0)		C-4
4	C	53.89	-		
5	CH	53.37	1.56		
6	CH_2_	21.73	2.14 †/1.23 †		
7	CH_2_	34.08	1.58 †/1.33 †		
8	C	43.06	-		
9	CH	48.43	1.05 dd		
10	C	37.50	-		
11	CH_2_	24.77	2.01 †/1.99 †		
12	CH	123.78	5.37 t (3.3)		
13	C	144.84	-		
14	C	41.24	-		
15	CH_2_	36.48	1.41 †/1.70 †		
16	CH	74.72	4.49 m		C-18
17	C	50.42	-		
18	CH	42.29	3.03 dd (14.0/4.0)		
19	CH_2_	47.92	2.28 dd (14.0/12.4) 1.05 dd (12.4/4.0)		
20	C	31.48	-		
21	CH_2_	36.62	1.93 †/1.15 ddd (15.0/12.4/4.4)		
22	CH_2_	32.15	1.92 †/1.75 ddd (15.0/15.0/4.4)		
23	C	182.74	-		
24	CH_3_	14.41	1.26 s		C-3, C-4, C-5
25	CH_3_	17.43	1.26 s		C-1, C-5, C-9, C-10
26	CH_3_	17.93	0.77 s		C-7, C-8, C-9, C-14
27	CH_3_	27.44	1.37 s		C-13, C-14, C-15
28	C	177.13	-		-
29	CH_3_	33.50	0.88 s		C-19, C-20, C-21, C-30
30	CH_2_	25.14	0.96 s		C-19, C-20, C-21, C-29
(**B**)					
		**5**			
	**C/H** **Atom**	**δ_C_ ppm**	**DEPT** **135**	**δ_H_ ppm, *J* (Hz)**	**HMBC** **from H to C**
Glc_1_-C(3)-Agly	1′	103.53	CH	4.59 d (8.2)	C-3
	2′	74.84	CH	4.76 dd (8.2/9.2)	***C***OCH_3_
	3′	74.30	CH	3.67 t (9.2)	
	4′	79.78	CH	3.70 t (9.2)	
	5′	76.52	CH	3.43 m	
	6′	61.49	CH_2_	3.90 †/3.82 †	
*C*OCH_3_		173.06	C	-	
CO*C*H_3_		21.60		2.15 s	***C***OCH_3_
Glc_2_-C(4′)-Glc_1_	1″	104.62	CH	4.43 d (7.8)	C-4′
	2″	75.07	CH	3.22 dd (7.8/9.2)	
	3″	77.97	CH	3.37 dd (9.2/9.2)	
	4″	71.57	CH	3.27 dd (9.2/9.2)	
	5″	78.31	CH	3.33 †	
	6″	62.65	CH_2_	3.87 †/3.63 †	
Ara-C(28)-Agly	1‴	94.36	CH	5.57 d (4.1)	C-28
	2‴	71.54	CH	3.80 †	
	3‴	75.20	CH	3.86 †	
	4‴	67.62	CH	3.82 †	
	5‴	64.42	CH_2_	3.89 †/3.51 †	
Rha_2_-C(3′′′′)-Ara	1′′′′	101.26	CH	5.10 d (1.2)	C-3‴
	2′′′′	72.18	CH	3.85 †	
	3′′′′	72.50	CH	3.81 †	
	4′′′′	83.82	CH	3.55 t (9.2)	
	5′′′′	68.97	CH	3.72 m	
	6′′′′	18.26	CH_3_	1.28 d (6.3)	
Xyl-(C-4′′′′)-Rha_1_	1′′′′′	106.71	CH	4.52 d (7.8)	C-4′′′′
	2′′′′′	76.04	CH	3.32 †	
	3′′′′′	84.83	CH	3.41 t (8.8)	
	4′′′′′	70.08	CH	3.52 †	
	5′′′′′	67.21	CH_2_	3.88 †/3.22 †	
Rha_2_-(C2′′′′′)-Xyl	1′′′′′′	101.79	CH	5.26 d (1.5)	C-3′′′′′
	2′′′′′′	80.33	CH	3.94 dd (1.8/3.3)	C-1′′′′′
	3′′′′′′	71.54	CH	3.86 †	
	4′′′′′′	74.48	CH	3.36 t (9.8)	
	5′′′′′′	70.43	CH	3.98 m	
	6′′′′′′	18.16	CH_3_	1.24 d (6.3)	
Api-C(3′′′′′′)-Rha_2_	1′′′′′′′	112.53	CH	5.12 d (2.6)	C-2′′′′′′
	2′′′′′′′	78.02	CH	3.96 d (2.6)	
	3′′′′′′′	80.69	C	-	
	4′′′′′′′	75.16	CH_2_	3.97 d (9.8)/3.74 d (9.8)	
	5′′′′′′′	65.78	CH_2_	3.60 br s (2H)	

† Signal patterns are unclear due to overlapping. Abbreviations: Agly, aglycone; Ara, arabinose; Glc, glucose; Rha, rhamnose; Xyl, xylose; Api, apiose.

**Table 5:** (**A**). The ^1^H and ^13^C-NMR data of **5a** and **5b** (δ_H_ 600 MHz, δ_C_ 150 MHz; MeOD). (**B**). The 1H and 13C-NMR data of **5a** (sugar moiety) (δH 600 MHz, δC 150 MHz; MeOD).

(**A**)						
		**5a**		**5b**		
**C/H** **Atom**	**DEPT** **135**	**δ_C_ ppm**	**δ_C_ ppm, *J* (Hz)**	**δ_C_ ppm**	**δ_C_ ppm, *J* (Hz)**	
1	CH_2_	44.98	2.12 dd (12.8/1.8)/1.28 †	45.01	2.14 dd (14.0/1.9)/1.30 †	
2	CH	71.80	4.31 m	71.40	4.33 m	
3	CH	86.96	4.10 d (3.6)	87.10	4.12 d (3.7)	
4	C	53.93	-	53.91	-	
5	CH	53.23	1.63 †	53.27	1.65 †	
6	CH_2_	21.95	1.61 †/1.23 †	21.83	1.65 †/1.25 †	
7	CH_2_	34.06	1.60 †/1.34 †	34.07	1.63 †/1.31 †	
8	C	43.07	-	43.04	-	
9	CH	48.76	1.67 †	48.34	1.68 dd (11.3/6.5)	
10	C	37.51	-	37.53	-	
11	CH_2_	24.77	1.99 †	24.76	2.01 †/1.96 †	
12	CH	123.78	5.39 t (3.6)	123.38	5.34 dd “t” (3.2)	
13	C	144.86	-	145.41	-	
14	C	41.32	-	41.18	-	
15	CH_2_	36.50	1.72 †/1.11 †	36.26	1.88 †/1.35 †	
16	CH	74.72	4.49 m	75.53	4.46 dd (3.2/3.2)	
17	C	50.43	-	49.98	-	
18	CH	42.29	3.04 dd (14.0/4.0)	42.35	3.05 dd (14.2/3.7)	
19	CH_2_	47.89	2.29 dd (14.0/14.0)/1.07 dd (14.0/4.0)	47.91	2.28 dd (14.2/14.2)/1.04 dd (12.7/2.9)	
20	C	31.48	-	31.54	-	
21	CH_2_	36.61	1.93 †/1.15 m	36.73	1.90 †/1.17 m	
22	CH_2_	32.13	1.93 †/1.76	32.65	1.91 †/1.78 ddd (17.7/12.7/4.1)	
23	C	184.57	-	182.17	-	
24	CH_3_	14.55	1.39 s	14.36	1.38 s	
25	CH_3_	17.52	1.29 s	17.40	1.30 s	
26	CH_3_	17.99	0.78 s	17.94	0.83	
27	CH_3_	27.51	1.38 s	27.55	1.39 s	
28	C	177.15	-	176.18	-	
29	CH_3_	33.51	0.90 s	33.60	0.90 s	
30	CH_2_	25.18	0.97 s	25.25	0.99 s	
(**B**)						
			**5a**		**5a**	
	**C/H** **Atom**	**DEPT** **135**	**δ_C_ ppm**	**δ_H_ ppm, *J* (Hz)**		
Glc_1_-C(3)-Agly	1′	CH	101.73	4.42 d (7.8)	104.83	4.427 d (8.0)
	2′	CH	76.08	3.30 †	75.02	3.30 dd (8.0/9.0)
	3′	CH	76.45	3.42 dd (9.0/9.0)	76.15	3.52 dd (9.0/9.0)
	4′	CH	80.16	3.62 dd (9.0/9.0)	80.20	3.63 dd (9.0/9.0)
	5′	CH	74.99	3.30 †	76.47 *	3.34 †
	6′	CH_2_	61.34	3.89/3.84	61.59	3.90 dd (12.0/3.5) 3.83 dd (12.0/2.2)
Glc_2_-C(4′)-Glc_1_	1″	CH	104.75	4.42 d (7.8)	104.74	4.43 d (8.0)
	2″	CH	76.15 *	3.24 dd (7.8/9.0)	75.13	3.25 dd (8.0/9.0)
	3″	CH	77.95	3.36 t (9.0)	77.96	3.39 dd “t” (9.0)
	4″	CH	71.46	3.32 †	71.50	3.34 †
	5″	CH	78.24	3.66 m	78.27	3.40 ddd (9.0/5.6/2.0)
	6″	CH_2_	62.53	3.89 †/3.67 dd (12.0/5.3)	62.53	3.88 dd (12.0/2.0) 3.67 dd (12.0/5.6)
Ara-C(28)-Agly	1‴	CH	94.33	5.57 d (4.0)		
	2‴	CH	71.46	3.32 †		
	3‴	CH	75.29	3.30 †		
	4‴	CH	67.55	3.83 †		
	5‴	CH_2_	64.34	3.90 †/3.53 †		
Rha_1_-C(2′′′)-Ara	1′′′′	CH	101.28	5.11 d (1.6)		
	2′′′′	CH	72.20	3.85 dd (1.6/3.39		
	3′′′′	CH	72.50	3.92–3.78 †		
	4′′′′	CH	83.76	3.56 dd (9.5/9.5)		
	5′′′′	CH	69.01	3.70 dq (9.5/6.1)		
	6′′′′	CH_3_	18.28	1.29 d (6.1)		
Xyl-(C-4′′′′)-Rha_1_	1′′′′′	CH	106.68	4.53 d (7.6)		
	2′′′′′	CH	76.08	3.32 †		
	3′′′′′	CH	84.75	3.42 t (9.5)		
	4′′′′′	CH	70.03	3.53 m		
	5′′′′′	CH_2_	67.22	3.87 †/3.23 dd (11.8/10.0)		
Rha_2_-C(2′′′′′)-Xyl	1′′′′′′	CH	104.71	5.27 d (1.6)		
	2′′′′′′	CH	80.16	3.94 dd (1.6/3.2)		
	3′′′′′′	CH	71.69	3.85 †		
	4′′′′′′	CH	74.48	3.36 dd (9.5/9.5)		
	5′′′′′′	CH	70.31	3.98 m		
	6′′′′′′	CH_3_	18.17	1.25 d (6.1)		
Api-C(3′′′′′′)-Rha_2_	1′′′′′′′	CH	112.46	5.15 d (2.7)		
	2′′′′′′′	CH	78.15	3.97 d (2.7)		
	3′′′′′′′	C	80.72	-		
	4′′′′′′′	CH_2_	75.14	3.97 d (9.7)/3.75 d (9.7)		
	5′′′′′′′	CH_2_	65.81	3.61 br s		

† Signal patterns are unclear due to overlapping. (*) 2β,3β,16α-trihydroxy-olean-12-en-23,29-dioic acid (zanhic acid). Abbreviations: Agly, aglycone; Ara, arabinose; Glc, glucose; Rha, rhamnose; Xyl, xylose; Api, apiose.

**Table 6:** α-Amylase and α-glucosidase inhibitory activity data.

Compounds and Fractions †	Antidiabetic Activity			
	α-Amylase Inhibitory Activity ^a^		α-Glucosidase Inhibitory Activity ^a^	
	IC50 µg/mL	µg Acarbose Equivalent/mg Extract	IC50 µg/mL	% Inhibition (50 µg/mL)
1	NA	35.18 ± 9.07	NA	NA
3	>100	11.87 ± 6.16	NA	NA
4	>100	45.76 ± 9.91	NA	NA
5	>100	21.24 ± 7.63	>50	24.06 ± 0.68
5a	>100	4.90 ± 13.30	>50	26.52 ± 2.30
5b	>100	37.83 ± 3.30	>50	5.212 ± 3.90
TD-Extract †	>100	7.05 ± 2.45	NA	NA
TD-G †	NA	NA	NA	NA
TD-F5 †	>100	3.09 ± 3.52	NA	NA
TD-C1 †	>100	12.36 ± 5.35	NA	NA
TZ-6–7 †	>100	6.66 ± 14.62	NA	NA
TZ-8–9 †	>100	31.15 ± 7.94	NA	NA
TZ-11 †	>100	20.92 ± 9.50	>50	6.211 ± 4.05
Acarboseb ^b^	62.92 ± 1.84	-	22.88 ± 0.52	80.53 ± 1.02

† Fractions coded as TD-X belong to *Teucrium davaeanum*, while fractions coded as TZ-X belong to *T. zanonii*. TZ-6–7, TZ-8–9 and TZ-11 are the fractions of methanolic extract of *T. zanonii*. TZ- were rich in compound **1**, TZ-8–9 were rich in compounds **2**, **3** and **4**, while 11 was rich in compound **5**. ^a^ Values expressed herein are means ± SEMs of three parallel measurements. *p* < 0.05. NA: not active. ^b^ Reference compound. Stock concentrations of purified compounds used for activity assays were 1000, 500, 250, and 125 µg/mL, while the extract was tested at 4000, 2000, 1000, and 500 µg/mL.

**Table 7:** The essential oil compositions of *Teucrium davaeanum* and *T. zanonii*.

LRI	Compound Name	Relative Percentage Amount (%)	
		*T. davaeanum*	*T. zanonii*
982	α-pinene	2.3	6.9
986	α-thujene	-	0.1
1030	Camphene	-	0.1
1077	β-pinene	9	19.5
1091	Sabinene	-	0.3
1132	Myrcene	0.3	0.7
1168	Limonene	1.5	2.5
1179	β-phellandrene	0.2	0.2
1241	p-cymene	0.2	0.2
1340	1-octenyl acetate	0.2	0.2
1406	1-octen-3-ol	-	0.2
1409	trans-linalool oxide	-	0.1
1417	α-cubebene	0.2	-
1434	Nerol oxide	-	0.1
1441	Bicycloelemene	1.3	0.5
1453	α-copaene	0.2	0.2
1459	Dihydroedulane II	0.3	0.3
1481	β-bourbonene	0.2	0.2
1501	Linalyl acetate	0.2	0.4
1511	Linalool	1.5	1.6
1536	β-ylangene	0.4	0.2
1541	α-bergamotene	0.2	0.1
1544	Pinocarvone	0.9	
1545	Bornyl acetate		0.3
1549	β-elemene		0.3
1550	α-guaiene	0.4	
1554	β-copaene	0.3	0.2
1559	Nopinone	0.2	0.2
1562	β-caryophyllene	3.7	0.3
1565	*E*-γ-bisabolene		3.3
1571	Aromadendrene	0.1	0.3
1602	Thuja-3-en-10-al	1	0.1
1608	Alloaromadendrene	0.2	1
1611	*trans*-pinocarvyl formate	0.8	0.2
1621	*trans*-pinocarveol	0.9	0.6
1628	β-guaiene	0.8	
1634	α-humulene	0.3	0.2
1642	*trans*-pinocarvyl acetate		0.3
1643	*cis*-verbenyl acetate	0.3	
1647	γ-humulene		0.4
1648	Epizonarene	0.2	1.3
1650	Myrtenyl acetate	0.4	0.2
1657	γ-terpinyl acetate	0.9	0.4
1661	Zonarene	0.7	1.1
1664	γ-guaiene	0.3	
1665	Borneol	-	0.3
1674	Germacrene D	31.4	0.1
1681	α-muurolene	-	13.4
1682	Valencene	0.4	-
1684	β-selinene	-	0.2
1688	Verbenone	-	0.1
1697	Bicyclogermacrene	15.9	0.2
1707	Carvone	0.2	5.3
1712	δ-cadinene	1.4	0.2
1719	γ-cadinene	0.2	0.8
1727	7-*epi*-α-selinene	0.1	0.4
1753	Myrtenol	0.7	0.1
1787	Cuparene	0.1	0.8
1792	Calamenene	0.7	0.1
1879	α-calacorene	0.1	0.1
1892	1,5-epoxy-salvial-4(14)-ene	-	0.1
1899	Epicubebol	0.3	-
1949	Longifolene aldehyde	-	0.1
1959	Caryophyllene oxide	0.7	0.2
1986	Dihydro-β-ionone	-	0.1
1987	Gleenol	0.3	-
2008	τ-cadinol acetate	0.2	0.1
2028	Cubenol	0.4	-
2029	1-*epi*-cubenol	-	0.1
2033	Hedycaryol	0.2	0.1
2040	Globulol	0.1	-
2043	Guaiol	0.2	-
2047	Viridiflorol	0.1	-
2067	Hexahydrofarnesyl acetate	1.0	0.2
2085	Spathulenol	3.4	0.1
2105	oxo-7,8-dihydro-β-ionol	-	9.2
2106	nor-copaanone	0.1	-
2108	Nonanoic acid	0.3	0.1
2125	τ-cadinol	0.7	0.3
2141	τ-muurolol	0.3	0.1
2151	Methyl palmitate	0.4	-
2152	Torreyol		0.3
2159	Carvacrol	0.8	0.1
2182	Guaia-6,10(14)-dien-4β-ol	-	1.1
2183	α-eudesmol	0.6	-
2189	Cadalene	1.0	1.3
2193	β-eudesmol	2.2	2.3
2212	Decanoic acid	0.5	0.4
2237	4-oxo-α-ylangene	0.8	1
2242	Nootkatol	-	0.1
2326	10-hydroxy-calamenone	-	0.2
2362	10-nor-calamenen-10-one	-	0.1
2425	Dodecanoic acid	1.8	1.8
2501	Nootkatone	0.3	0.2
2626	Nonacosane	-	0.4
2670	Myristic acid	0.6	0.7
Total		99.1	88.2

LRI: Relative retention index calculated against *n*-alkanes; % calculated from FID data.

## Data Availability

The authors declare that the data supporting the findings of this study are available within the paper and its Supplementary Information Files. Should any raw data files be needed in another format, they are available from the corresponding authors upon reasonable request. Source data are provided with this paper.

## Supplementary Materials

The following supporting information can be downloaded at: https://www.mdpi.com/article/10.3390/molecules31122196/s1, Figure S1. Teucardoside (**1**); Figure S2. Positive ion ESI MS of Teucardoside (**1**): *m*/*z* 513 [M+Na]^+^; Figure S3. The ^1^H-NMR Spectrum of Teucardoside (**1**) (δH 600 MHz, CD_3_OD); Figure S4. The ^13^C-NMR Spectrum of Teucardoside (**1**) (δH 150 MHz, CD_3_OD); Figure S5. DEPT-135 of Teucardoside (**1**); Figure S6. The HMBC of Teucardoside (**1**); Figure S7. Poliumoside (**2**); Figure S8. Negative ion ESI MS of Poliumoside (**2**); Figure S9. ^1^H-NMR Spectrum of Poliumoside (**2**) (δ_H_ 600 MHz, CD_3_OD); Figure S10. ^13^C-NMR and DEPT-135 Spectra of TD-D1 (Poliumoside) (δ_C_ 150 MHz, CD_3_OD); Figure S11. 3-O-Methyl-Poliumoside (**3**); Figure S12. Negative ion ESI MS of 3-O-Methyl-Poliumoside (**3**); Figure S13. ^1^H-NMR Spectrum of 3-O-Methyl-poliumoside (**3**) (δ_H_ 500 MHz, CD_3_OD); Figure S14. The ^13^C-NMR and DEPT-135 Spectra of 3-O-Methyl-poliumoside (**3**) (δ_C_ 125 MHz, CD_3_OD); Figure S15. Vicenin-2 (**4**); Figure S16. Positive ion ESI MS of Vicenin-2 (**4**); Figure S17. The ^1^H-NMR Spectrum of Vicenin-2 (**4**) (δ_H_ 500 MHz, CD_3_OD); Figure S18. ^13^C-NMR Spectrum of Vicenin-2 (**4**) (δ_C_ 125 MHz, CD_3_OD); Figure S19. DEPT-135 of Vicenin-2 (**4**); Figure S20. Daveaenoside (**5**); Figure S21. Negative ion ESI MS of Daveaenoside (**5**); Figure S22. The ^1^H-NMR Spectrum of Davaeanoside (**5**) (δ_H_ 600 MHz, CD_3_OD); Figure S23. The ^13^C-NMR and DEPT-135 Spectra of Davaeanoside (**5**) (δ_C_ 150 MHz, CD_3_OD); Figure S24. The COSY of Davaeanoside (**5**); Figure S25. TOCSY of Davaeanoside (**5**); Figure S26.1. HSQC of Davaeanoside (**5**); Figure S26.2. HSQC of Davaeanoside (**5**); Figure S27. HSQC-TOCSY of Davaeanoside (**5**); Figure S28.1. HMBC of Davaeanoside (**5**) (Sapogenol moiety); Figure S28.2. HMBC of Davaeanoside (**5**) (Sugar moiety); Figure S28.3. HMBC of Davaeanoside (**5**) (Sugar moiety); Figure S29. NOESY of Davaeanoside (**5**); Figure S30. Prodaeaenoside (**5a**); Figure S31. Negative ion ESI-MS of Prodavaeanoside (**5a**); Figure S32. The ^1^H-NMR Spectrum of Prodavaeanoside (**5a**) (δ_H_ 500 MHz, CD_3_OD); Figure S33. The ^1^C-NMR Spectrum of Prodavaeanoside (**5a**) (δ_H_ 125 MHz, CD_3_OD); Figure S34. DEPT-135 of Prodavaeanoside (**5a**); Figure S35. COSY of Prodavaeanoside (**5a**); Figure S36. HSQC of Prodavaeanoside (**5a**); Figure S37. HMBC of Prodavaeanoside (**5a**); Figure S38. Deacetyl-Daveaenoside (**5b**); Figure S39. Negative ion ESI-MS of Deacetyl-davaeanoside (**5b**); Figure S40. The ^1^H-NMR Spectrum of Deacetyl-davaeanoside (**5b**) (δ_H_ 500 MHz, CD_3_OD); Figure S41. The ^13^C-NMR Spectrum of Deacetyl-davaeanoside (**5b**) (δ_H_ 125 MHz, CD_3_OD); Figure S42. DEPT-135 of Deacetyl-davaeanoside (**5b**); Figure S43. COSY of Deacetyl-davaeanoside (**5b**); Figure S44. HSQC of Deacetyl-davaeanoside (**5b**); Figure S45. HMBC of Deacetyl-davaeanoside (**5b**).
