# Supplementary material for: Comparative Phytochemical Studies on the Aerial Parts of Teucrium davaeanum Coss. and Teucrium zanonii Pamp
Source: Molecules. 2026 Jun 22;31(12):2196. doi: 10.3390/molecules31122196 (PMC13305427; doi:10.3390/molecules31122196)
Supplement: Supplementary file 1 [file molecules-31-02196-s001.zip › molecules-4359364-supplementary.pdf]

Supplementary Material

List of Supplementary Data

Figure S1. Teucardoside (**1**)....................1
Figure S2. Positive ion ESI MS of Teucardoside (**1**)....................1
Figure S3. The ^{1}H-NMR Spectrum of Teucardoside (**1**) (δH 600 MHz, CD_3OD)....................2
Figure S4. The ^{13}C-NMR Spectrum of Teucardoside (**1**) (δH 150 MHz, CD_3OD)....................2
Figure S5. DEPT-135 of Teucardoside (**1**)....................3
Figure S6. HMBC of Teucardoside (**1**)....................3
Figure S7. Poliumoside (**2**)....................4
Figure S8. **N**egative ion ESI-MS of Poliumoside (**2**)....................4
Figure S9. **The** ^{1}H-NMR Spectrum of Poliumoside (**2**) (δ_H 600 MHz, CD_3OD)....................5
Figure S10. ^{13}C-NMR and DEPT-135 Spectra of Poliumoside (**2**) (δ_C 150 MHz, CD_3OD)....................5
Figure S11. 3-*O*-Methyl**-**Poliumoside (**3**)....................6
Figure S12. Negative ion ESI-MS of 3-*O*-Methyl-Poliumoside (**3**)....................6
Figure S13. The ^{1}H-NMR Spectrum of 3-O-Methyl-Poliumoside (**3**) (δ_H 600 MHz, CD_3OD)....................7
Figure 14.The ^{13}C-NMR and DEPT-135 Spectra of 3-*O*-Methyl-Poliumoside (**3**)....................7
Figure S15. Vicenin-2 (**4**)....................8
Figure S16. Negative ion ESI-MS of Vicenin-2 (**4**)....................8
Figure S17. ^{1}H-NMR Spectrum of Vicenin-2 (4) (δ_H 600 MHz, CD_3OD)....................9
Figure S18. ^{13}C-NMR Spectrum of Vicenin-2 (4) (δ_C 150 MHz, CD_3OD)....................9
Figure S19. DEPT-135 of Vicenin-2 (**4**)....................10
Figure S20. Daveaenoside (**5**)**.**....................11
Figure S21. Negative ion ESI MS of Daveaenoside (**5**)....................11
Figure S22. The ^{1}H-NMR Spectrum of Daveaenoside (**5**).....................12
Figure S23. The ^{13}C-NMR and DEPT-135 Spectra of Davaeanoside (**5**) (δ_C 150 MHz, MeOD)....................12
Figure S24. COSY of Davaeanoside (**5**)....................12
Figure S25. TOCSY of Davaeanoside (**5**)....................13
Figure S26.1. HSQC of Davaeanoside (**5**)....................13
Figure S26.2. HSQC of Davaeanoside (**5**)....................14
Figure S27. HSQC-TOSY of Davaeanoside (**5**)....................14
Figure S28.1. HMBC of Davaeanoside (**5**) (Sapogenol moiety)....................15
Figure S28.2. HMBC of Davaeanoside (**5**) (Sugar moiety)....................15
Figure S28.3. HMBC of Davaeanoside (**5**)....................16
Figure S29. NOESY of Davaeanoside (**5**)....................16
Figure S30. Prodaveaenoside (**5a**)....................17

Figure S31. Negative ion ESI_MS of Prodavaeanoside (**5a**)........17
Figure S32. ^{1}H-NMR Spectrum of Prodavaeanoside (**5a**) (δ_H 500 MHz, MeOD)........17
Figure S33. The ^{13}C-NMR Spectrum of Prodavaeanoside (**5a**) (δ_C 125 MHz, MeOD)........18
Figure **S34.** DEPT-135 of Prodavaeanoside (**5a**)........18
Figure S35. COSY of Prodavaeanoside (**5a**)........19
Figure S36. HSQC of of Prodavaeanoside (**5a**)........19
Figure S37. HSQC of of Prodavaeanoside (**5a**)........20
Figure S38. Deacetyl-Daveaenoside (**5b**)........21
Figure S39. Negative ion ESI_MS of Deacetyl-davaeanoside (**5b**)........21
Figure S40. ^{1}H-NMR Spectrum of Deacetyl-davaeanoside (**5b**) (δ_H 500 MHz, MeOD)........22
Figure S41. The ^{13}C-NMR Spectrum of Deacetyl-davaeanoside (**5b**) (δ_C 125 MHz, MeOD)........22
Figure S42. DEPT-135 of Deacetyl-davaeanoside (**5b**)........23
Figure S43. COSY of Deacetyl-davaeanoside (**5b**)........23
Figure S44. HSQC of of Deacetyl-davaeanoside (**5b**)........24
Figure S45. HMBC of Deacetyl-davaeanoside (**5b**)........24

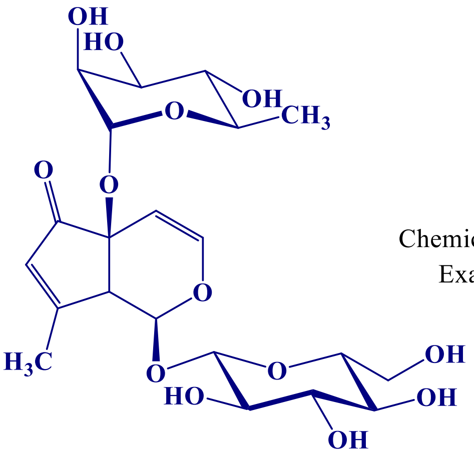

Chemical Formula: $C_{21}H_{30}O_{13}$
Exact Mass: 490.16864

Figure S1. Teucardoside (**1**)

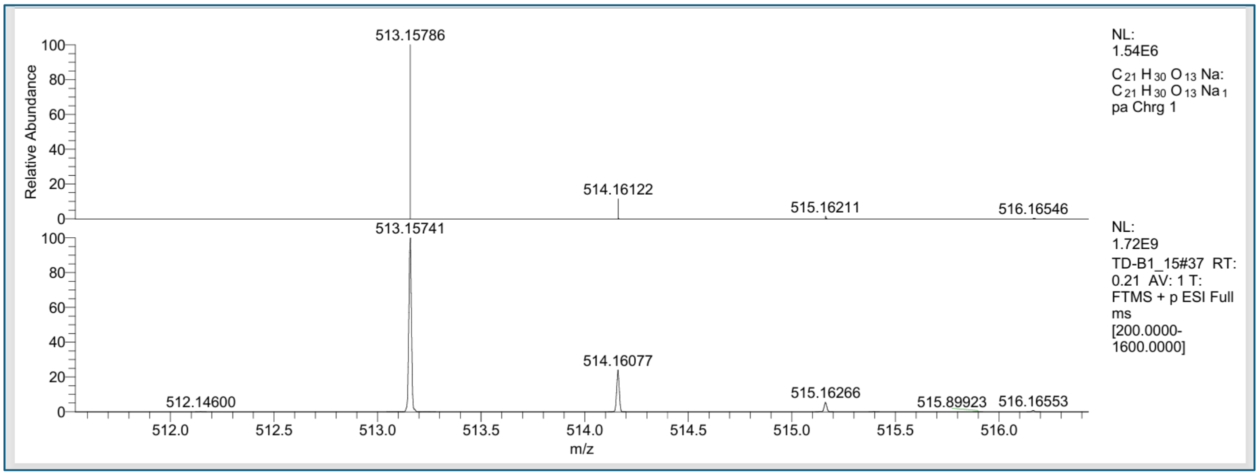

Figure S2. Positive ion ESI MS of Teucardoside (**1**): m/z 513 $[M+Na]^+$**.**

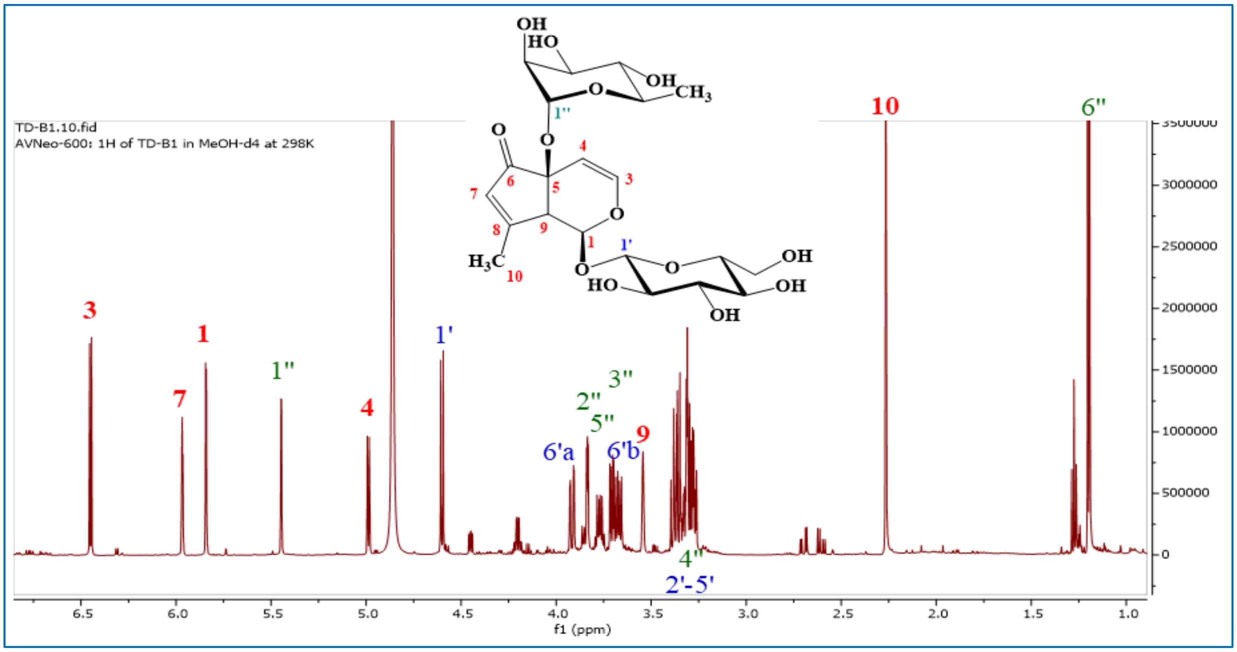

Figure S3. The ^{1}H-NMR Spectrum of Teucardoside (**1**) (δH 600 MHz, CD_3OD)

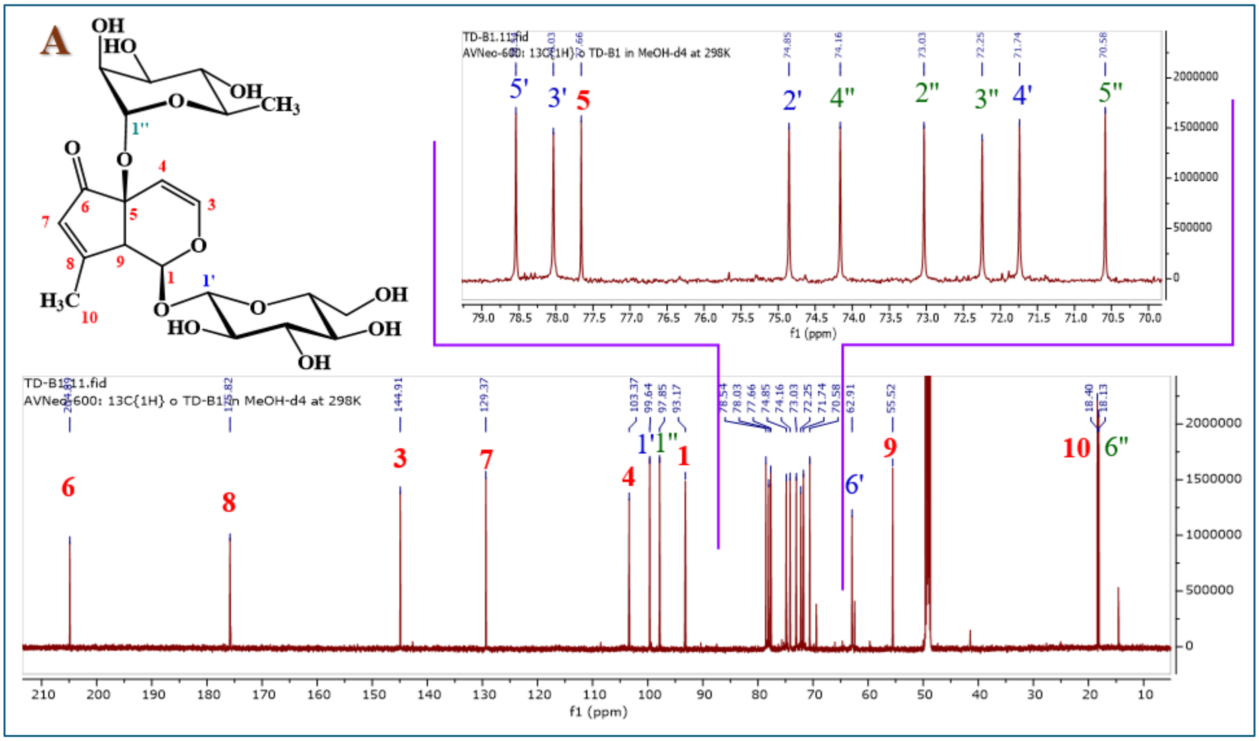

Figure S4. The ^{13}C-NMR Spectrum of Teucardoside (**1**) (δH 150 MHz, CD_3OD).

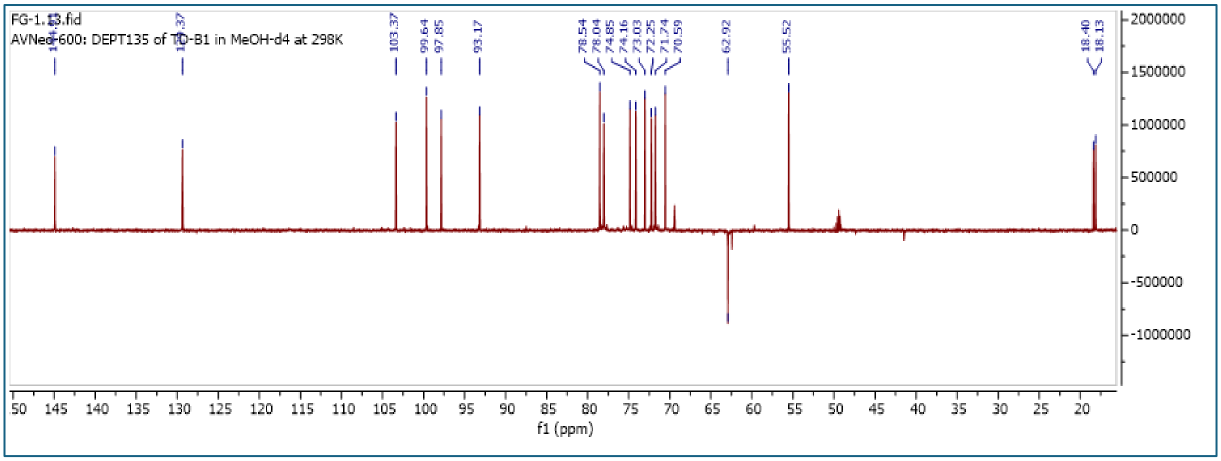

Figure S5. DEPT-135 of Teucardoside (**1**).

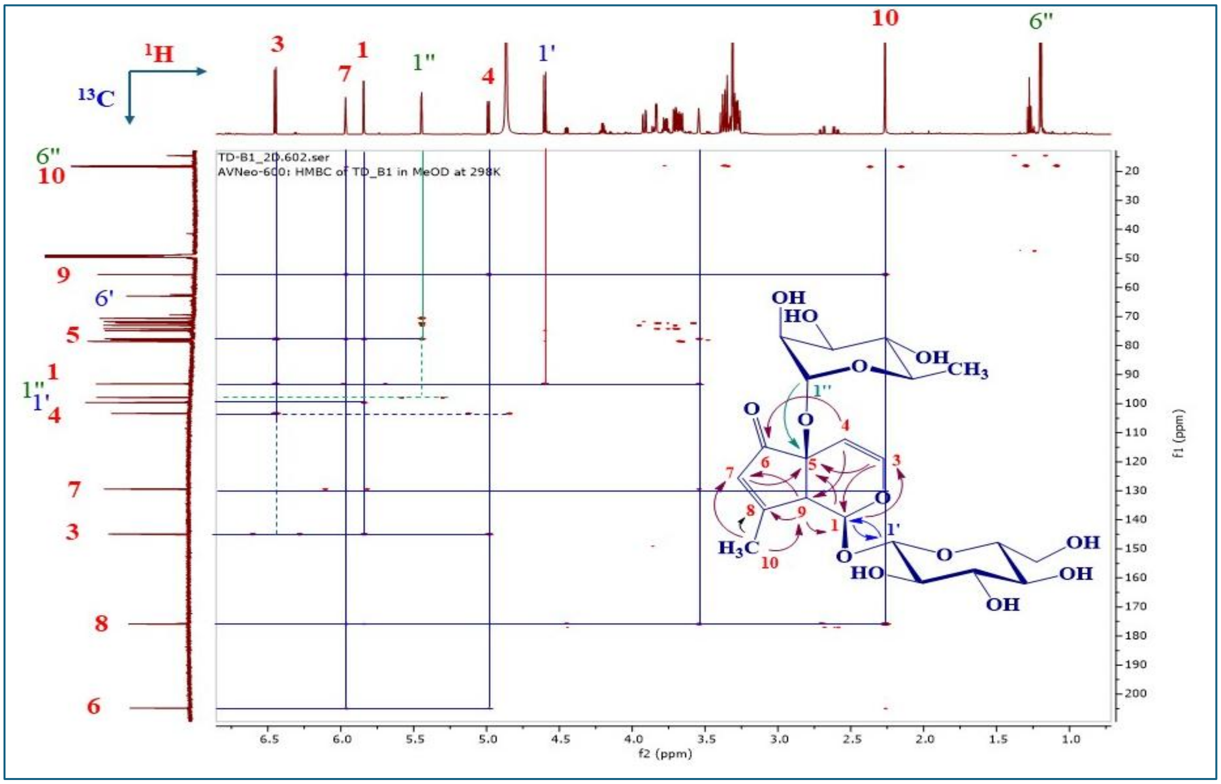

Figure S6. The HMBC of Teucardoside (**1**).

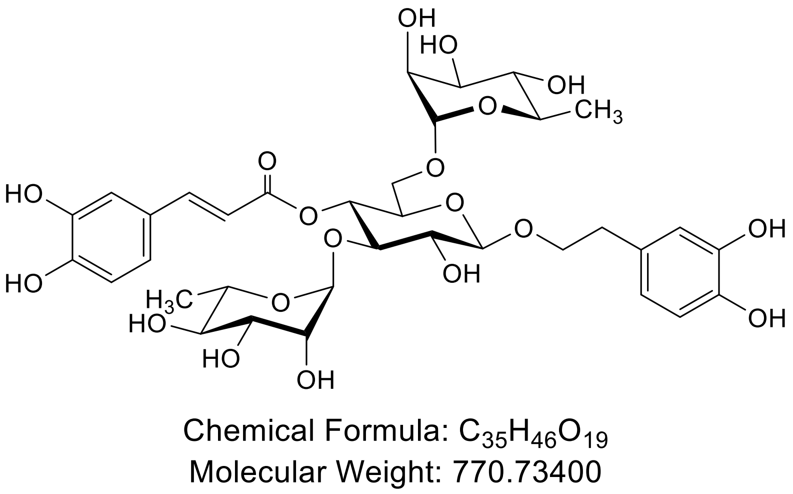

Figure S7. Poliumoside (**2**).

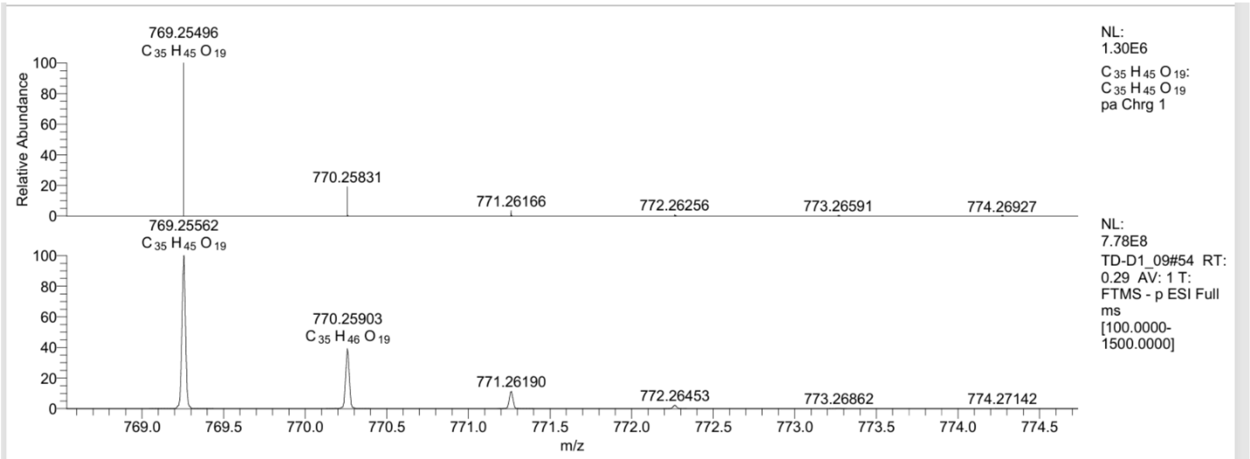

Figure S8. Negative ion ESI MS of Poliumoside (**2**).

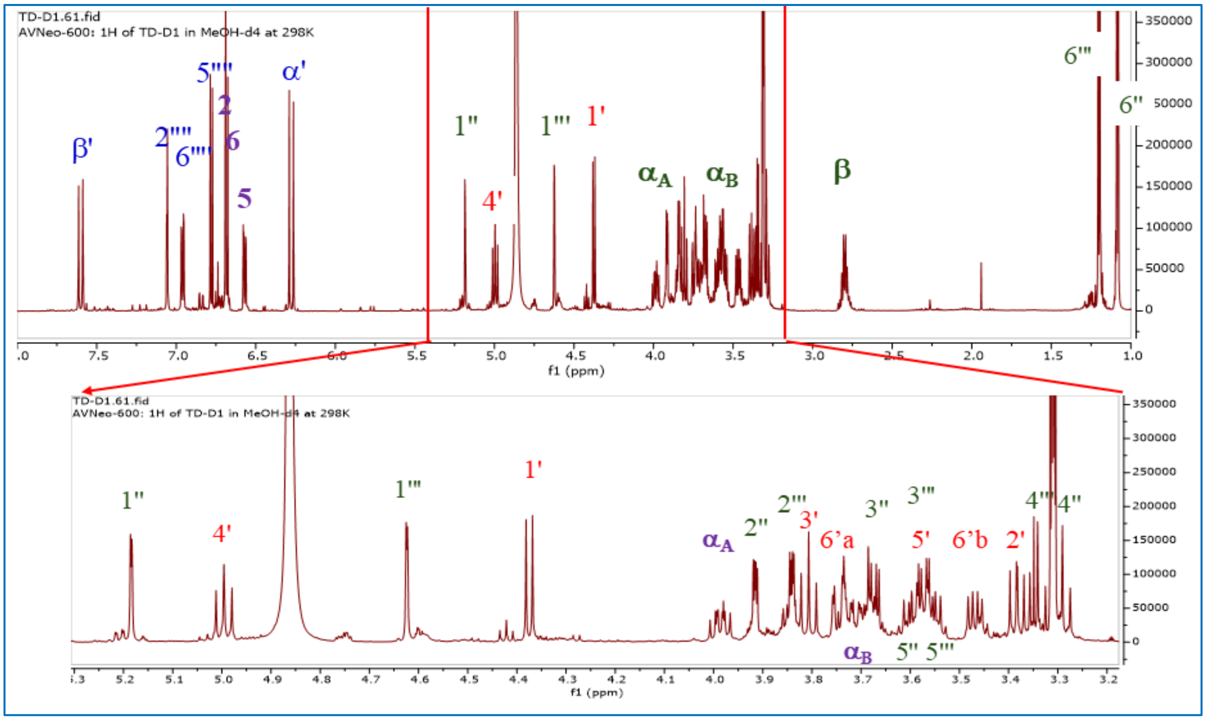

Figure S9. ^{1}H-NMR Spectrum of Poliumoside (**2**) (δ_H 600 MHz, CD_3OD)

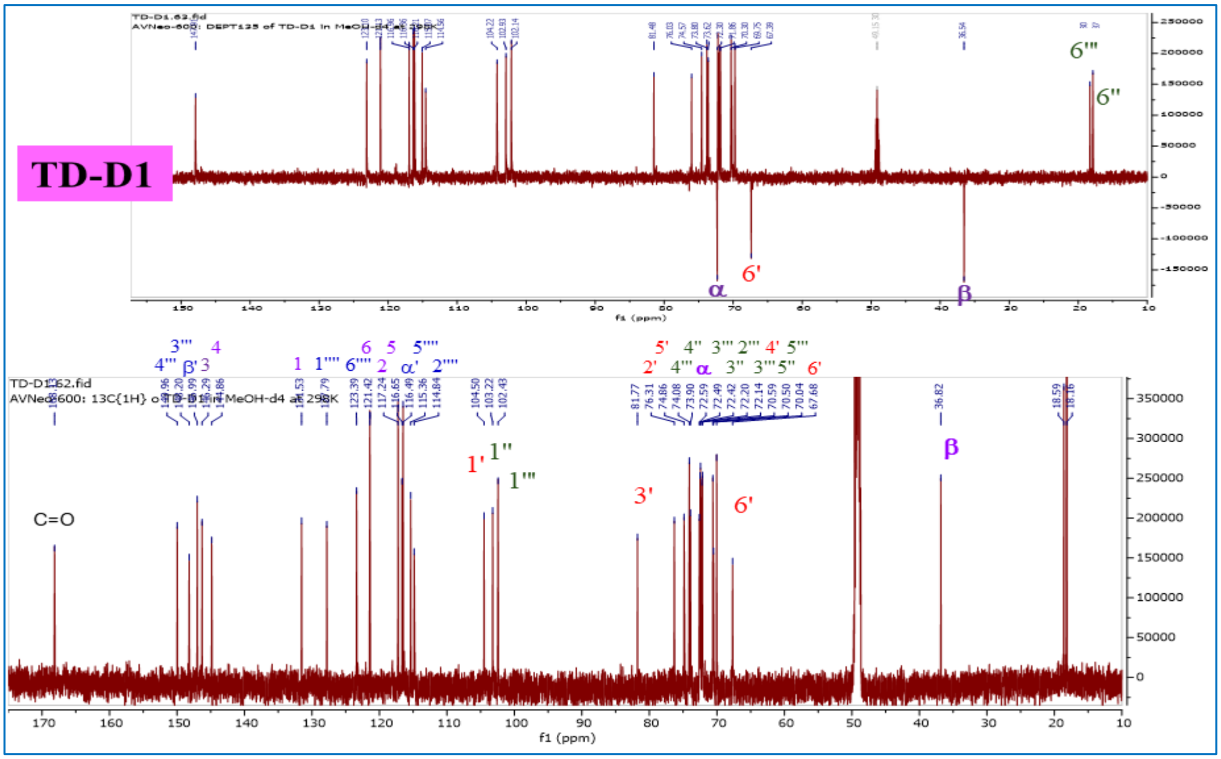

Figure S10. ^{13}C-NMR and DEPT-135 Spectra of **TD-D1 (Poliumoside)** (δ_C 150 MHz, CD_3OD)

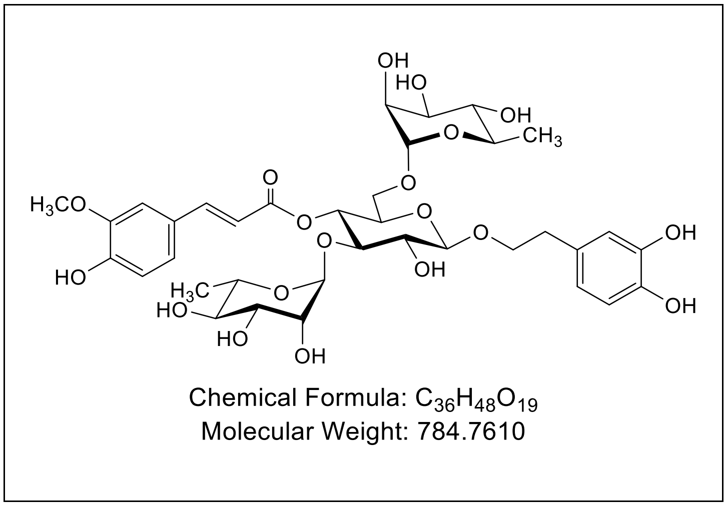

Figure S11. 3-*O*-Methyl-Poliumoside (**3**).

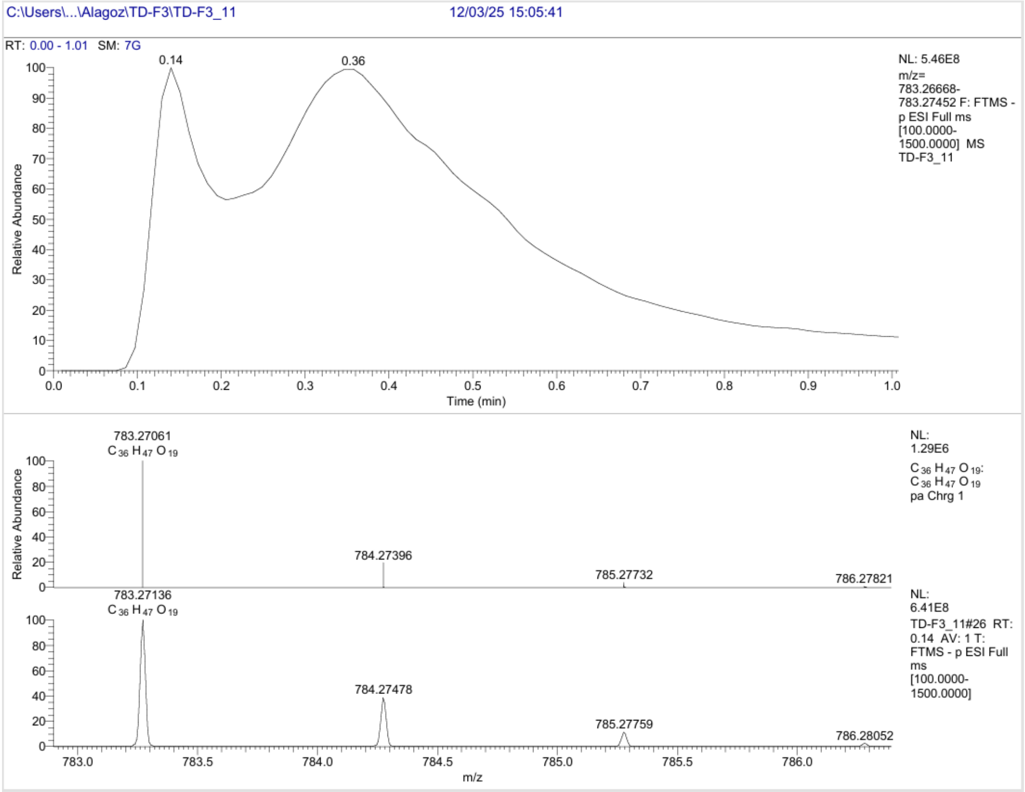

Figure S12. Negative ion ESI MS of 3-O-Methyl-Poliumoside (**3**).

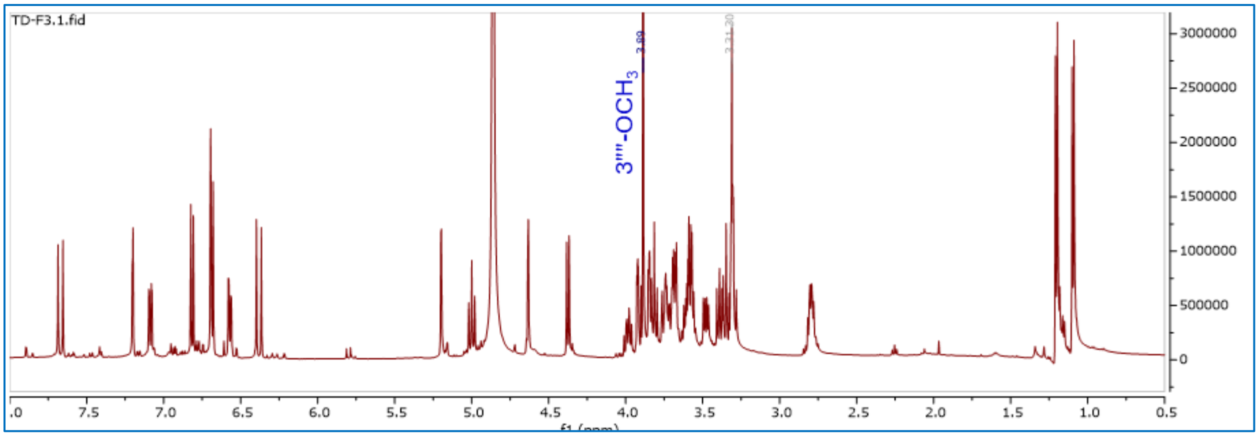

Figure S13. ^{1}H-NMR Spectrum of 3-*O*-Methyl-poliumoside (**3**) (δ_H 500 MHz, CD_3OD).

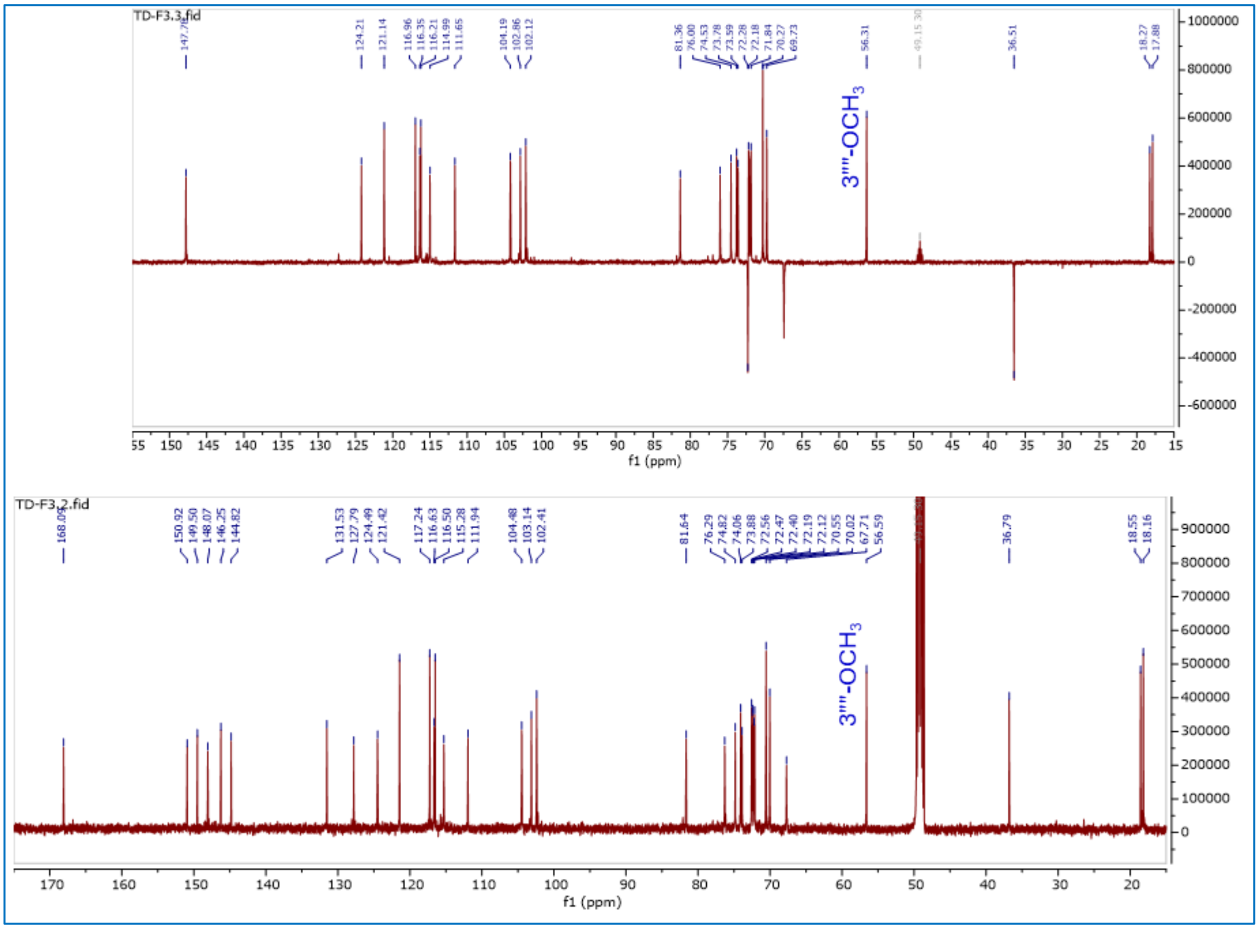

FigureS14. The ^{13}C-NMR and DEPT-135 Spectra of 3-*O*-Methyl-poliumoside (**3**)

(δ_C 125 MHz, CD_3OD).

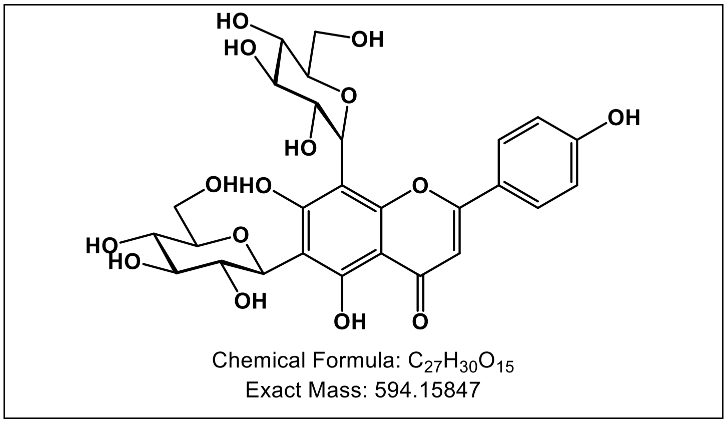

Figure S15. Vicenin-2 (**4**).

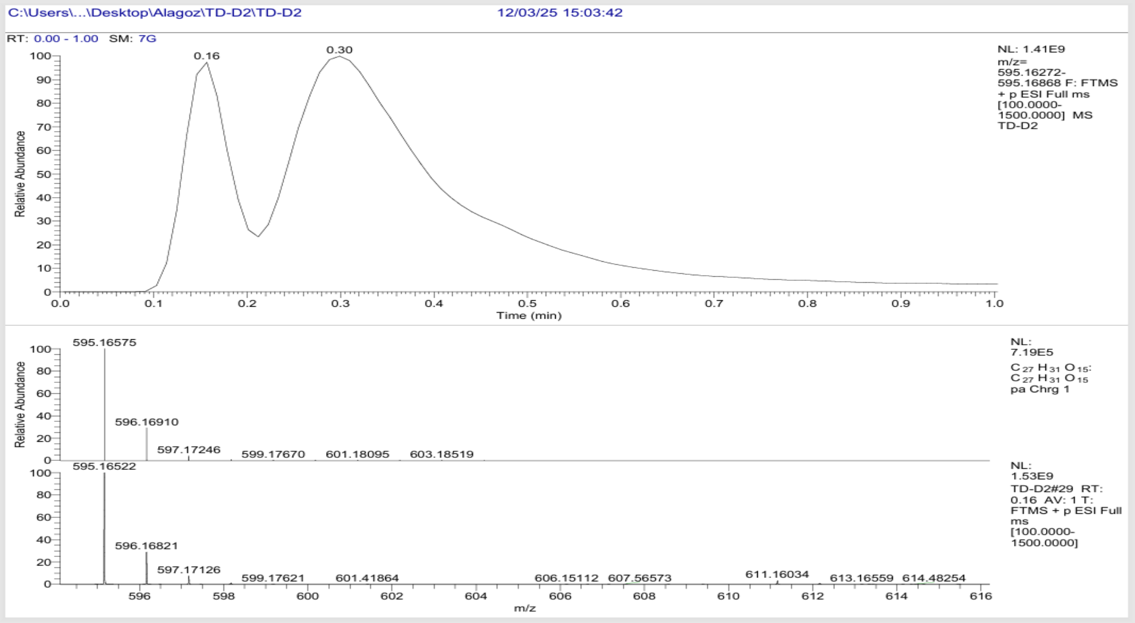

Figure S16. Positive ion ESI MS of **Vicenin-2** (**4**).

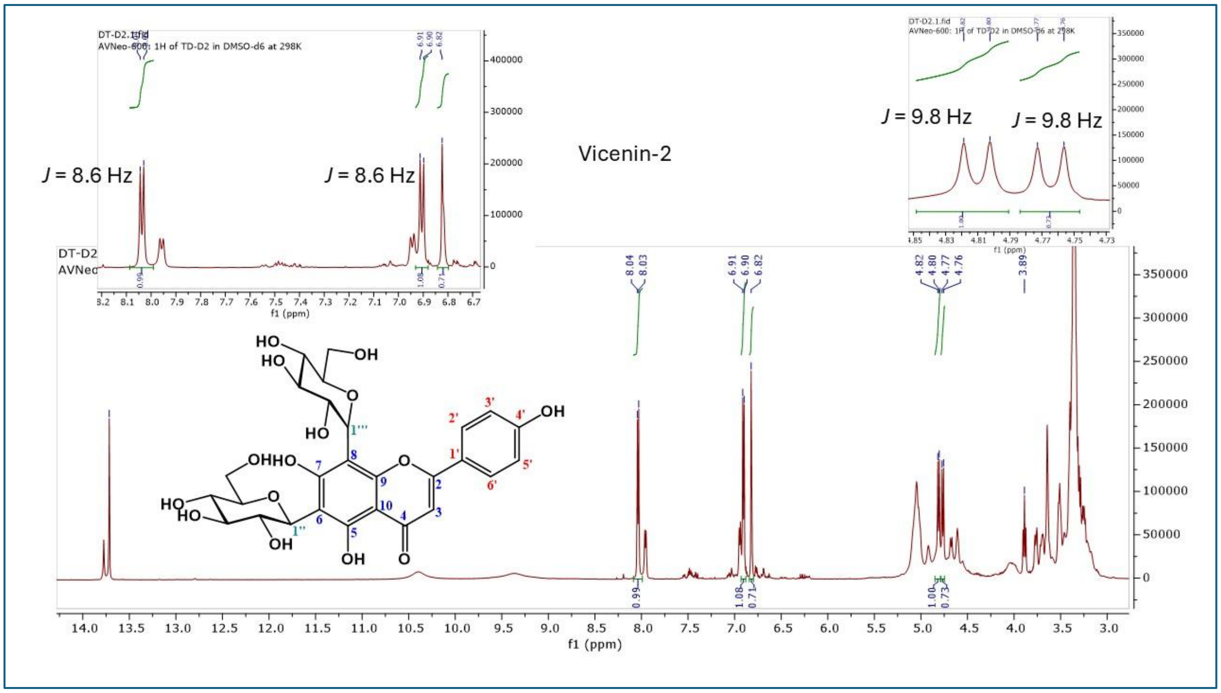

Figure S17. The ^{1}H-NMR Spectrum of Vicenin-2 (**4**) (δ_H 500 MHz, CD_3OD).

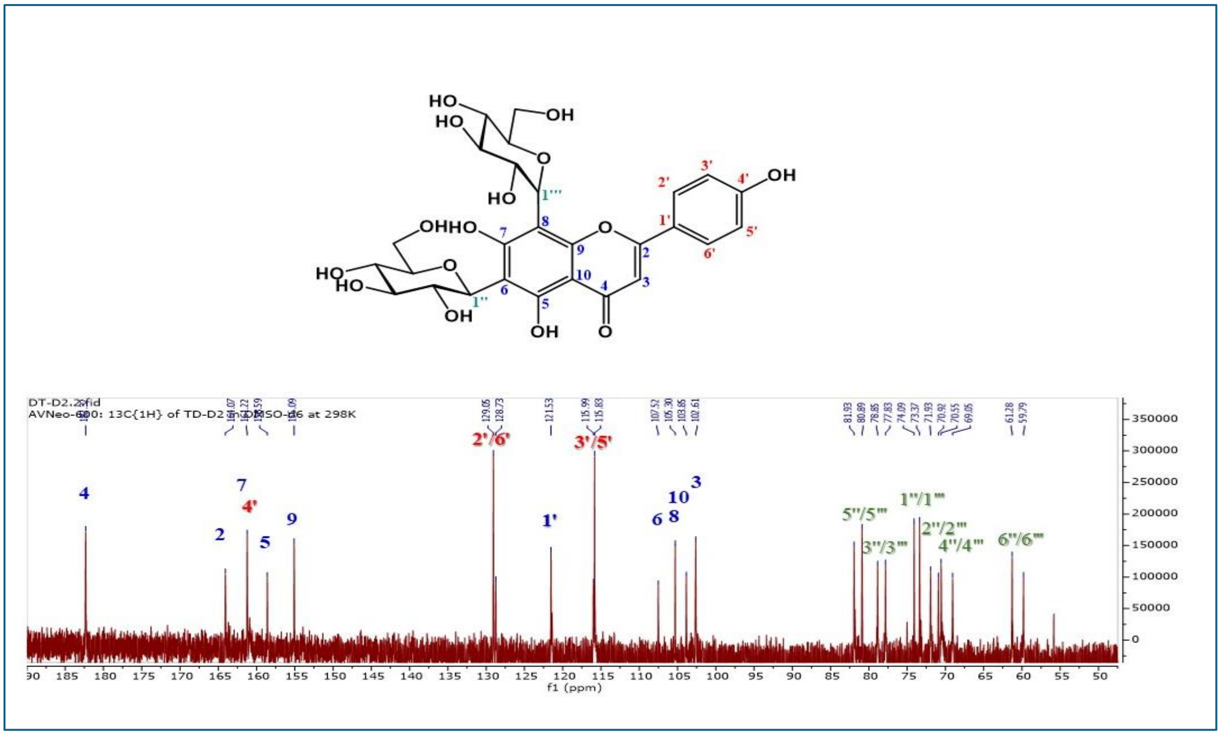

Figure S18. ^{13}C-NMR Spectrum of Vicenin-2 (**4**) (δ_C 125 MHz, CD_3OD).

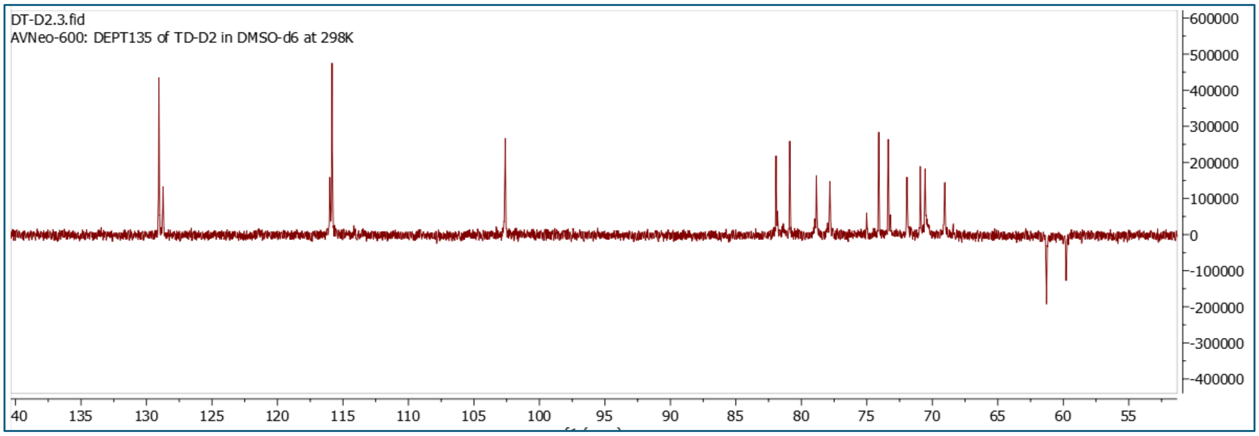

Figure S19. DEPT-135 of Vicenin-2 (**4**).

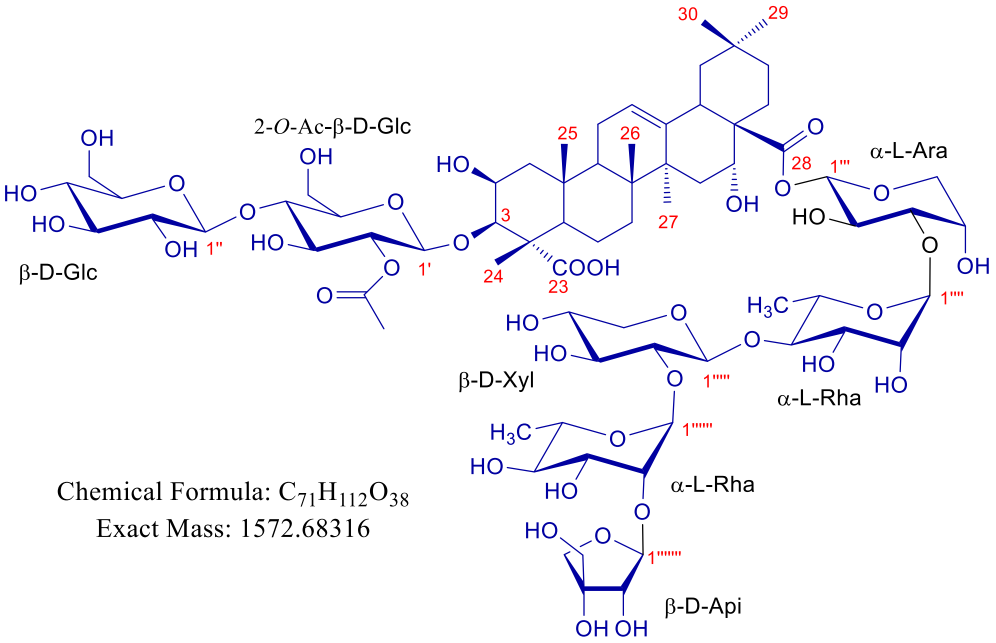

Figure S20. Daveaenoside (**5**).

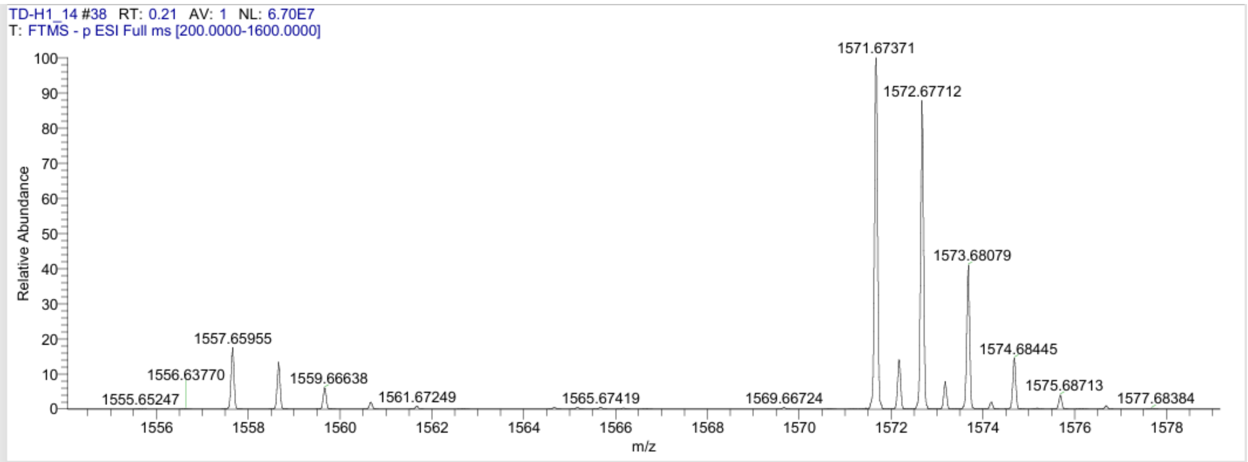

Figure S21: Negative ion ESI MS of Daveaenoside (**5**).

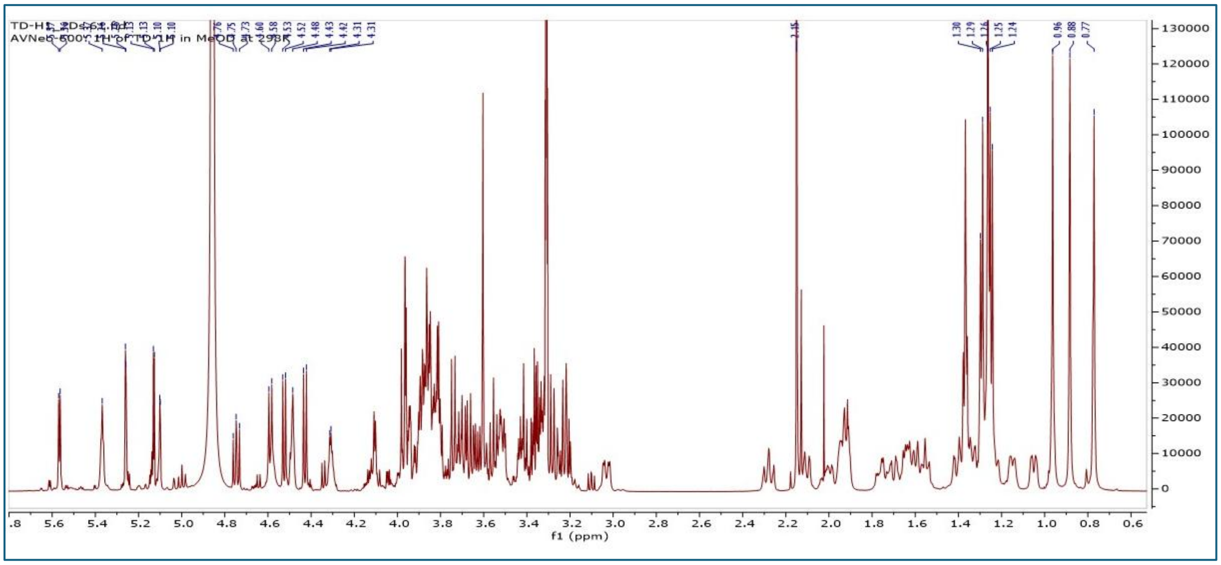

Figure S22. The ^{1}H-NMR Spectrum of Davaeanoside (**5**) (δ_H 600 MHz, CD_3OD).

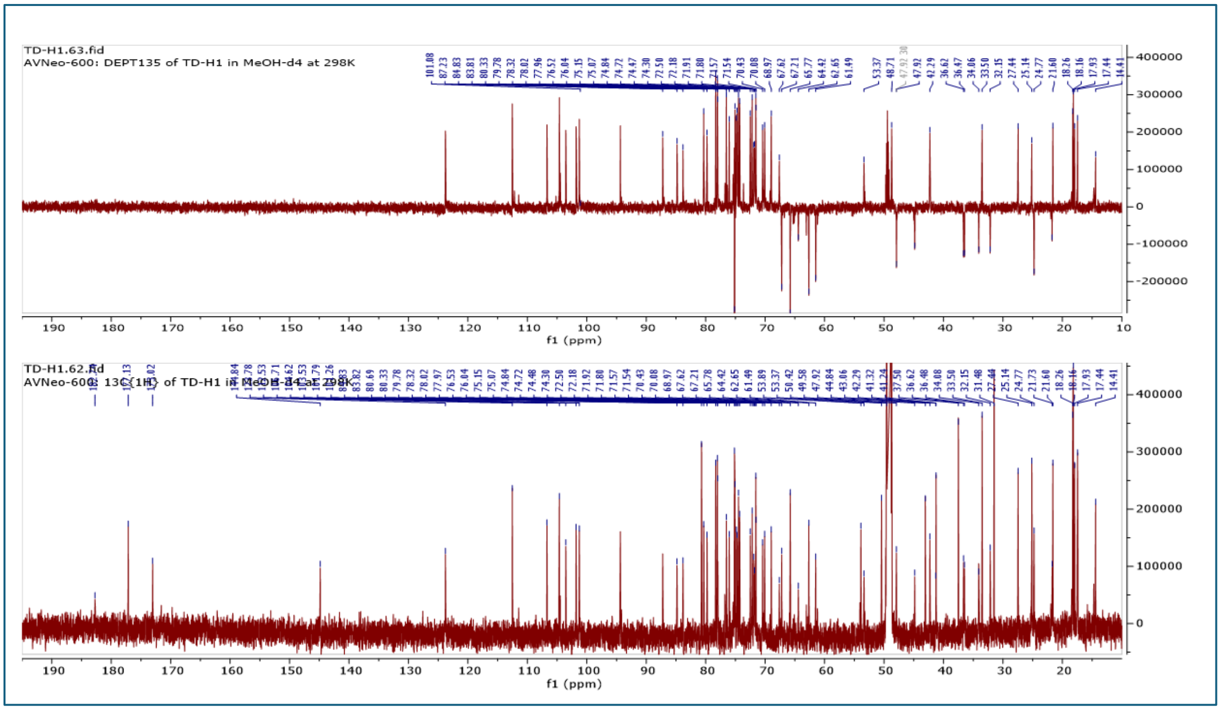

Figure S23. The ^{13}C-NMR and DEPT-135 Spectra of Davaeanoside (**5**) (δ_C 150 MHz, CD_3OD).

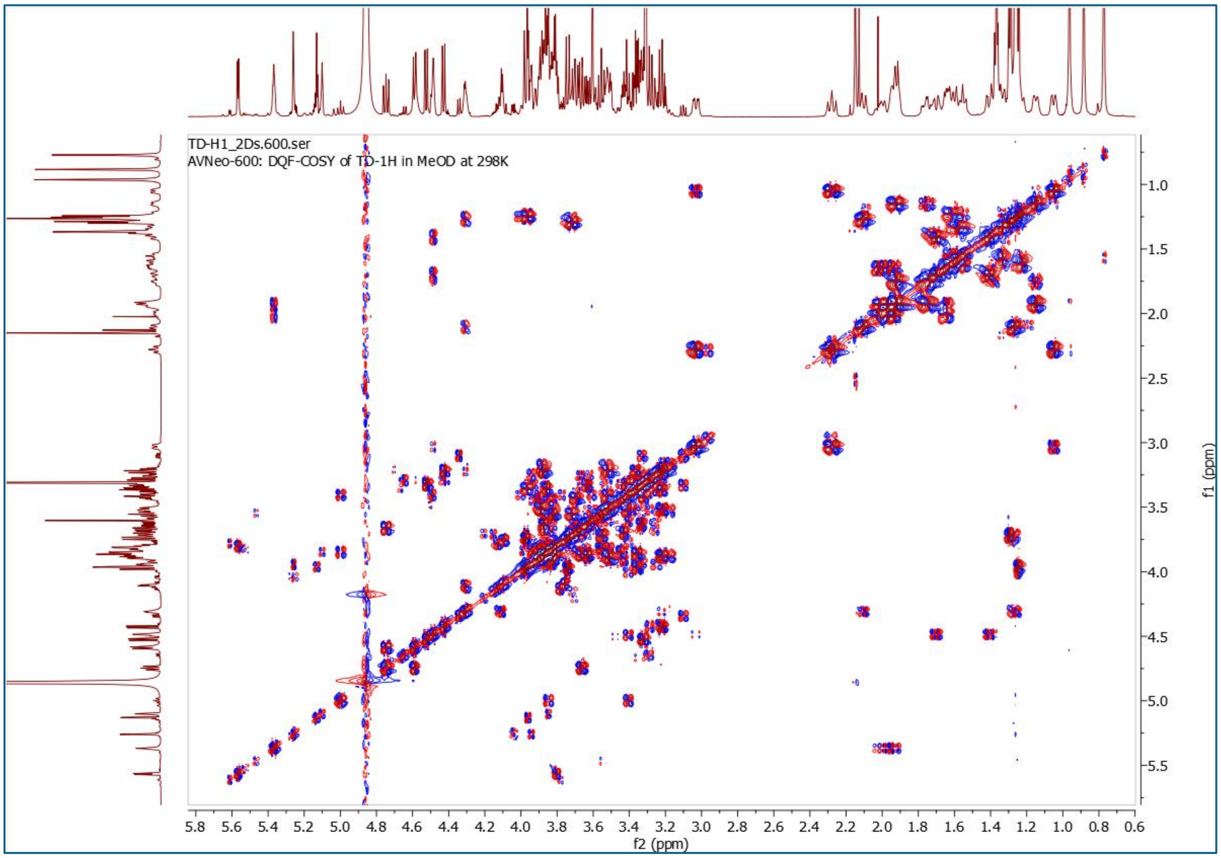

Figure S24. The COSY of Davaeanoside (**5**).

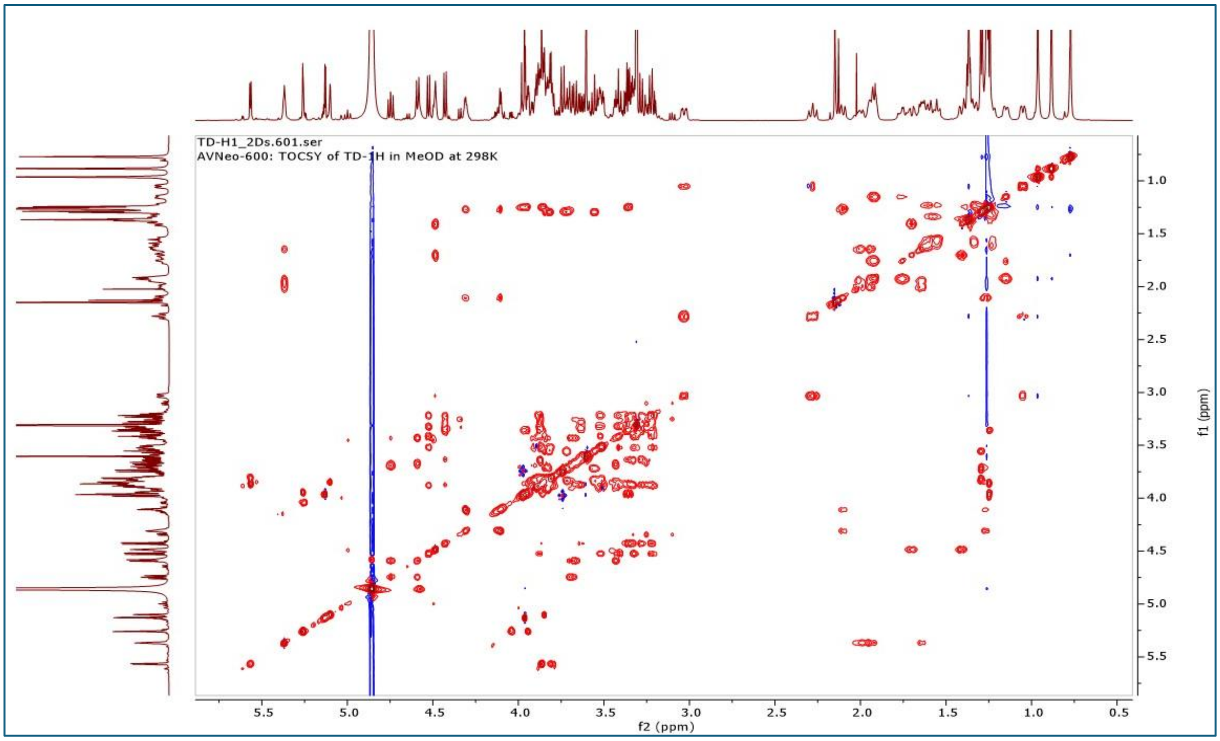

Figure S25. TOCSY of Davaeanoside (**5**).

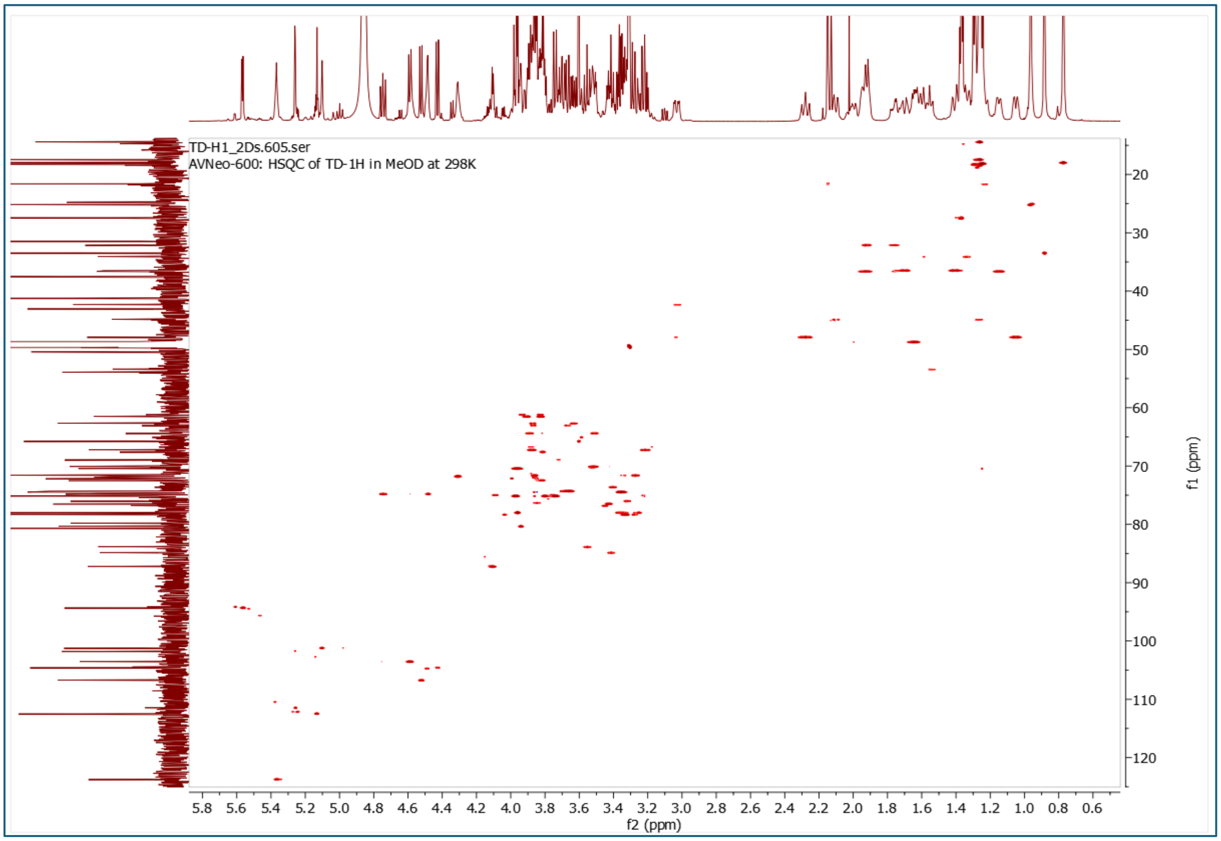

Figure S26.1. HSQC of Davaeanoside (**5**).

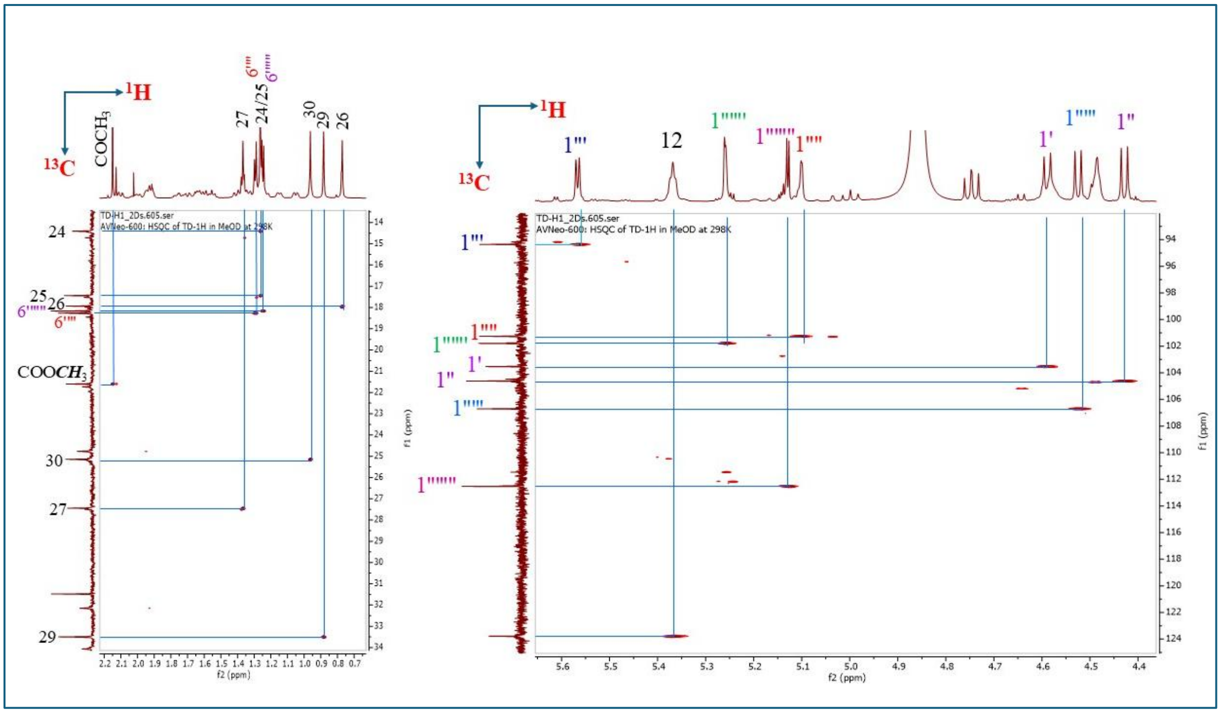

Figure S26.2. HSQC of Davaeanoside (**5**).

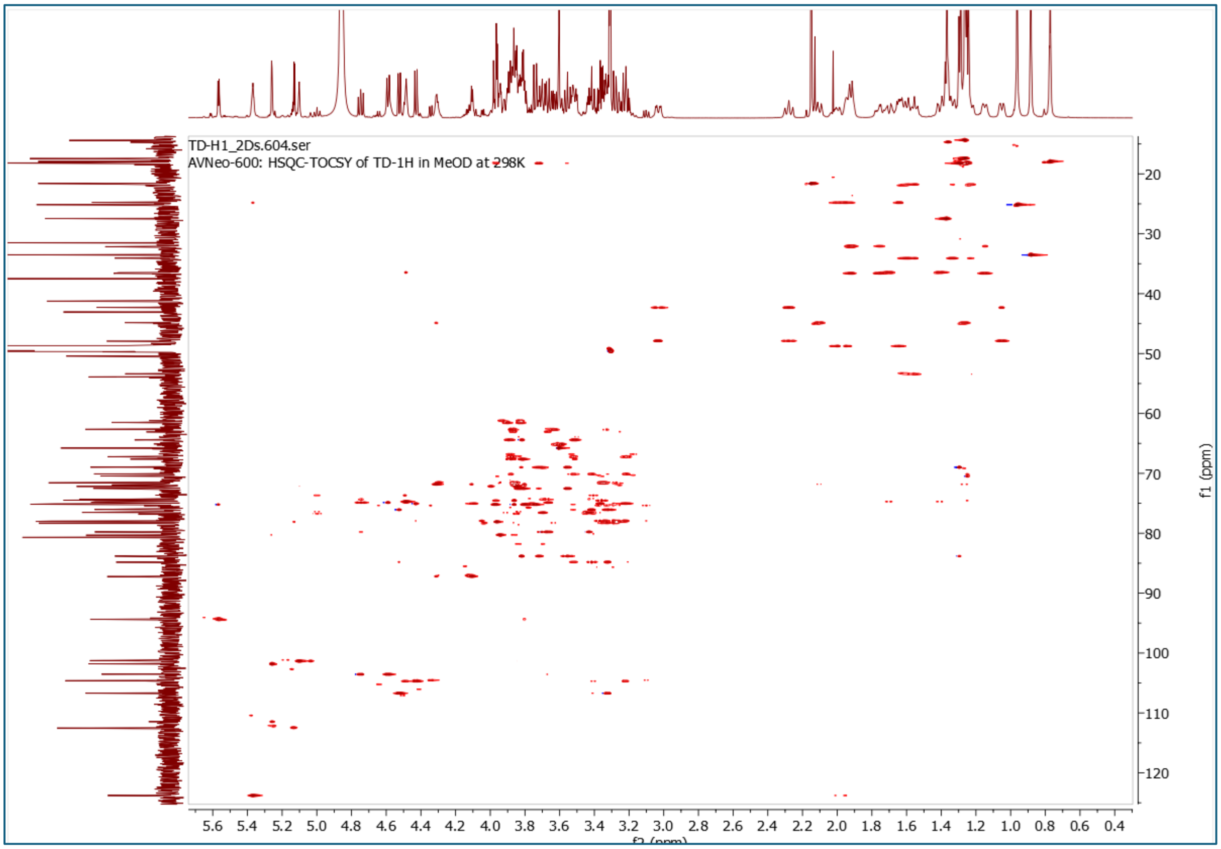

Figure S27. HSQC-TOCSY of Davaeanoside (**5**).

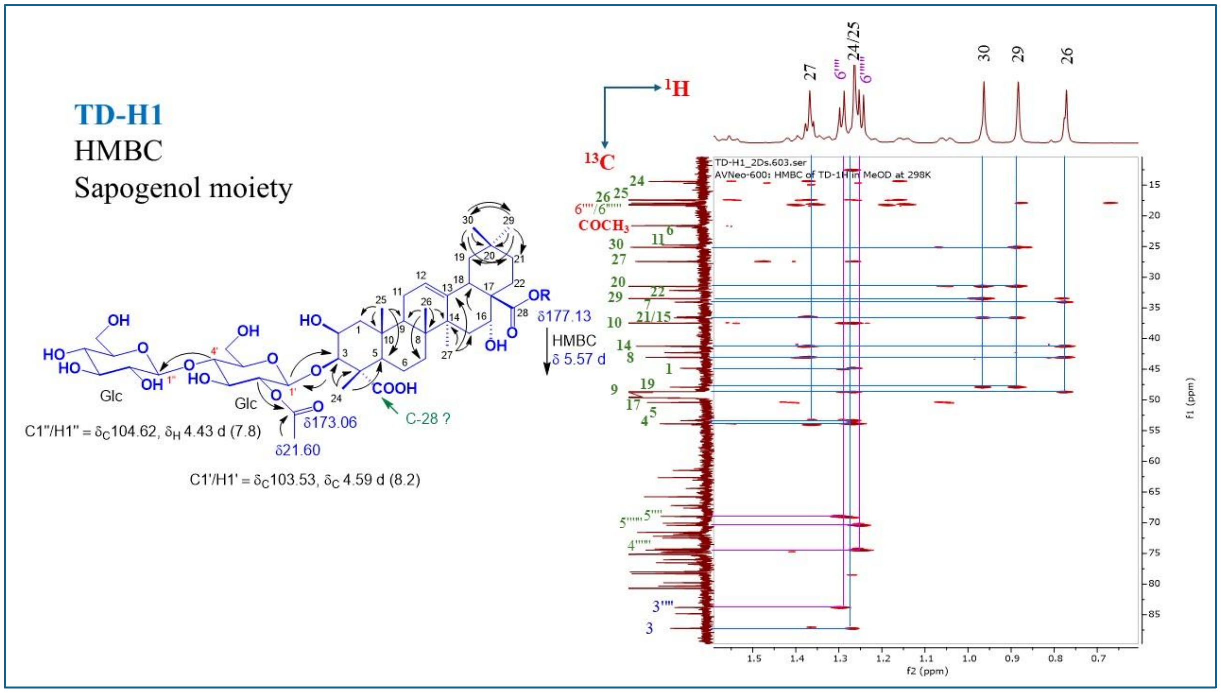

Figure S28.1. HMBC of Davaeanoside (**5**) (Sapogenol moiety).

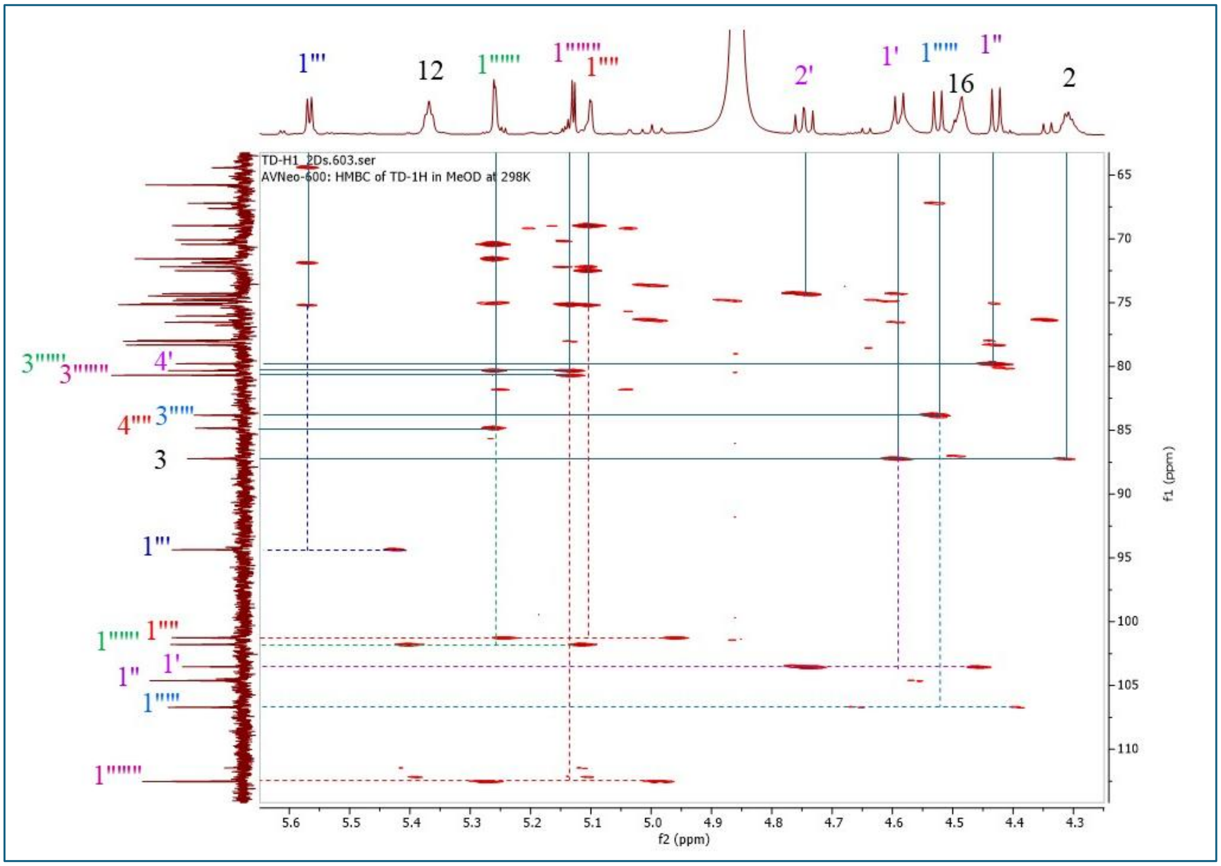

Figure S28.2. HMBC of Davaeanoside (**5**) (Sugar moiety).

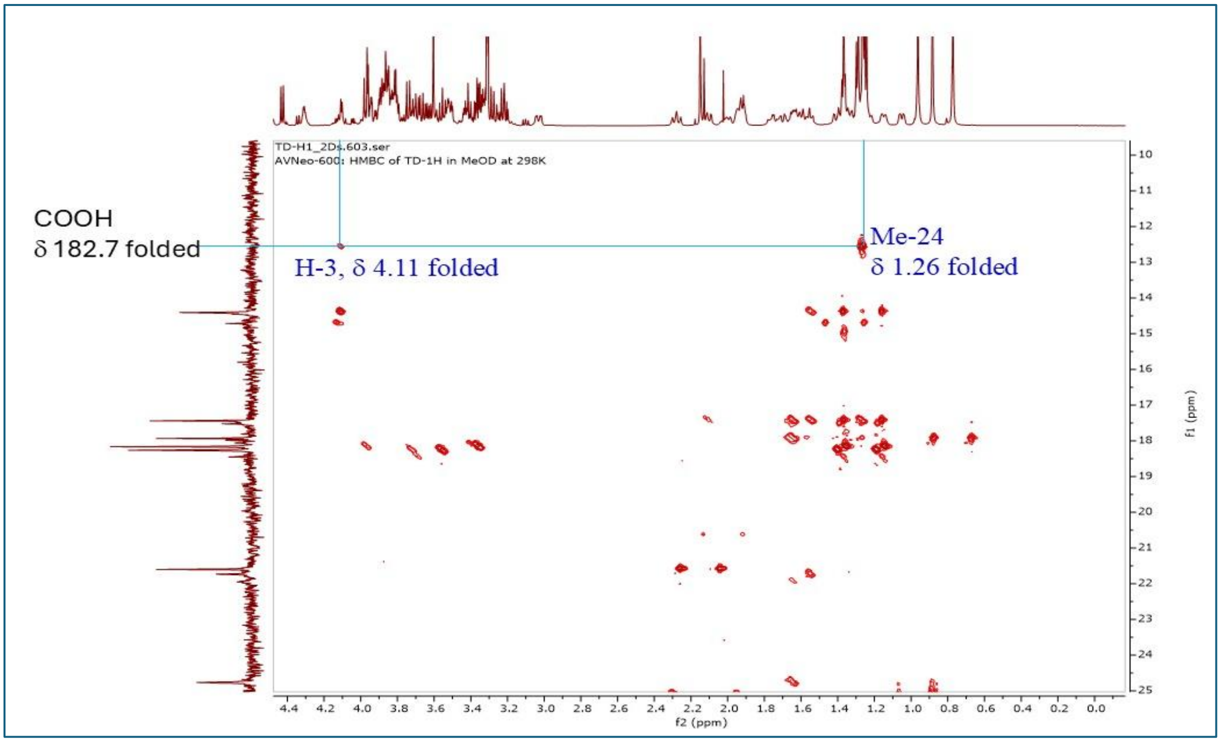

Figure S28.3. HMBC of Davaeanoside (**5**) (Sugar moiety).

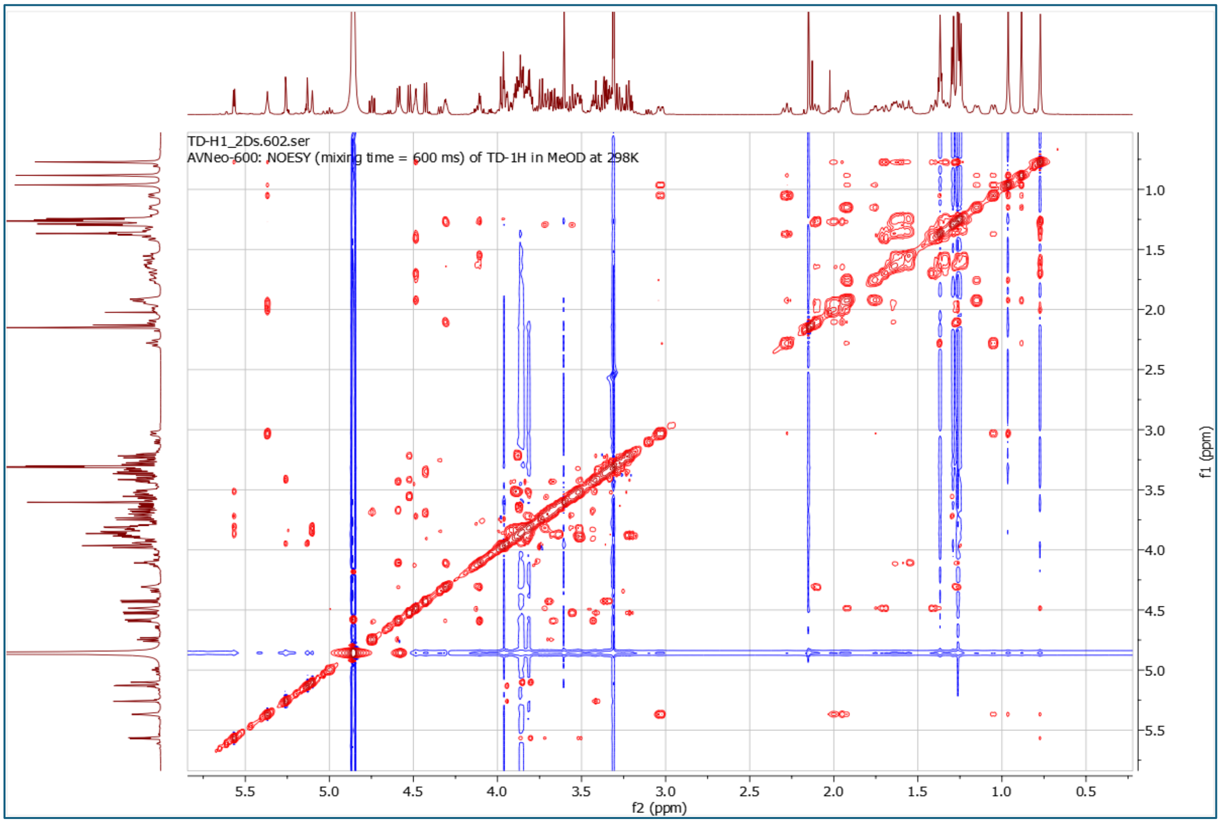

Figure S29. NOESY of Davaeanoside (**5**).

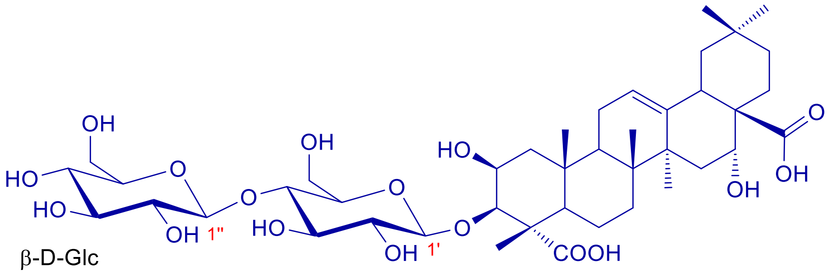

Chemical Formula: $C_{42}H_{66}O_{17}$
Exact Mass: 842.43000

Figure S30. Prodaeaenoside (**5a**)

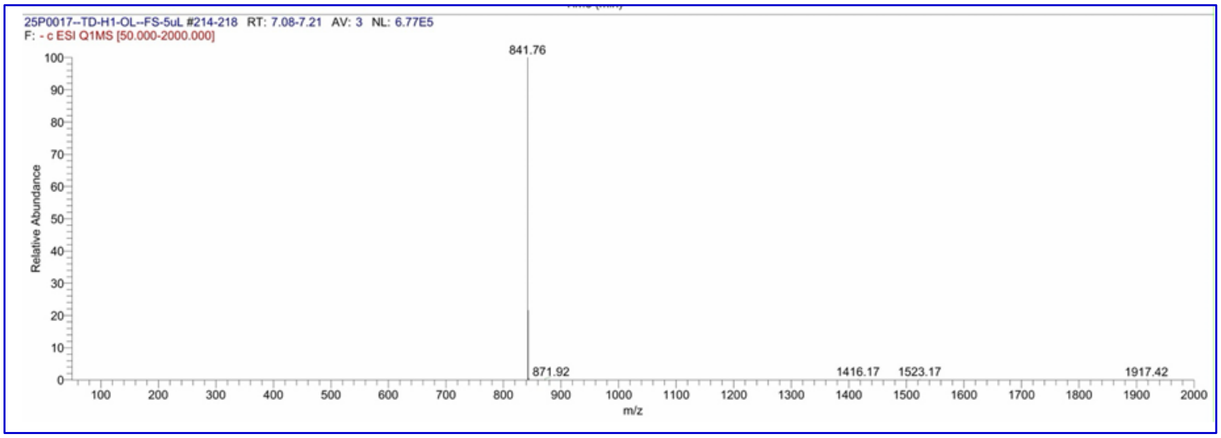

Figure S31. Negative ion ESI-MS of Prodavaeanoside (**5a**)

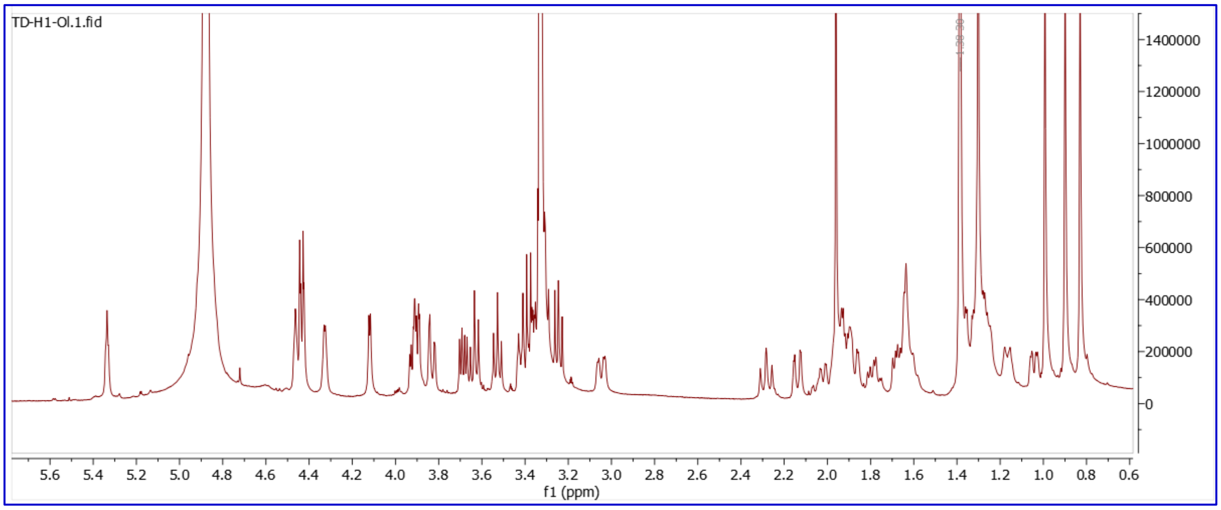

Figure S32. The ^{1}H-NMR Spectrum of Prodavaeanoside (**5a**) (δ_H 500 MHz, CD_3OD).

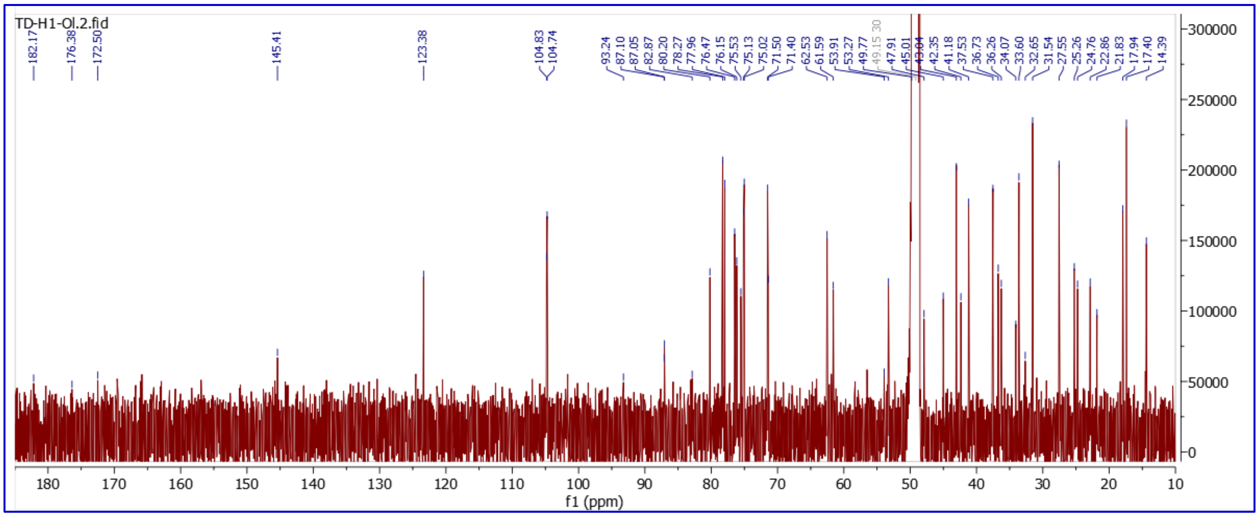

Figure S33. The ^{1}C-NMR Spectrum of Prodavaeanoside (**5a**) (δ_H 125 MHz, CD_3OD).

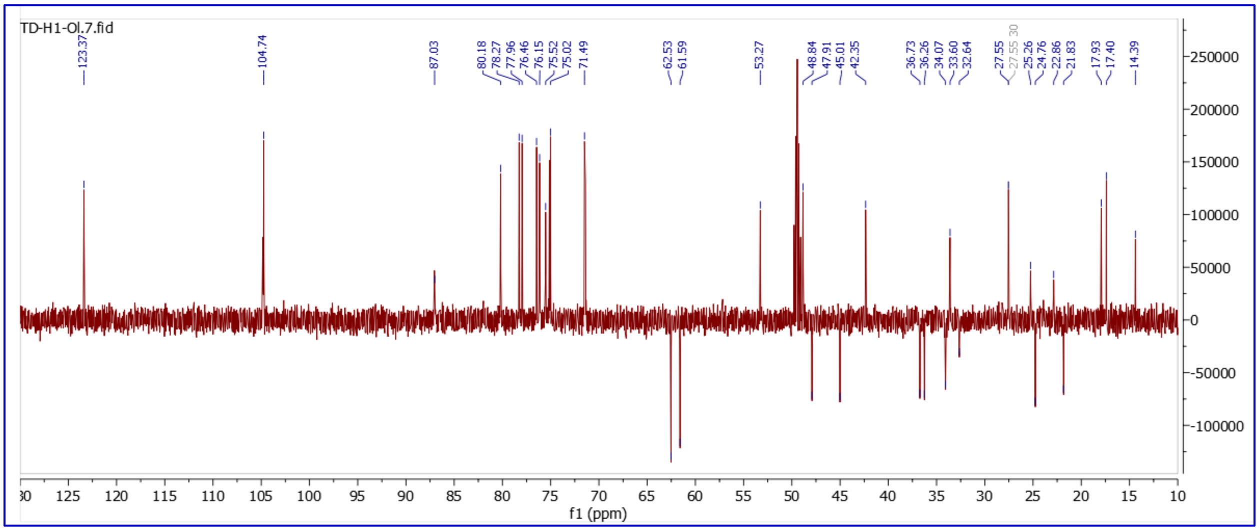

Figure S34. DEPT-135 of Prodavaeanoside (**5a**)

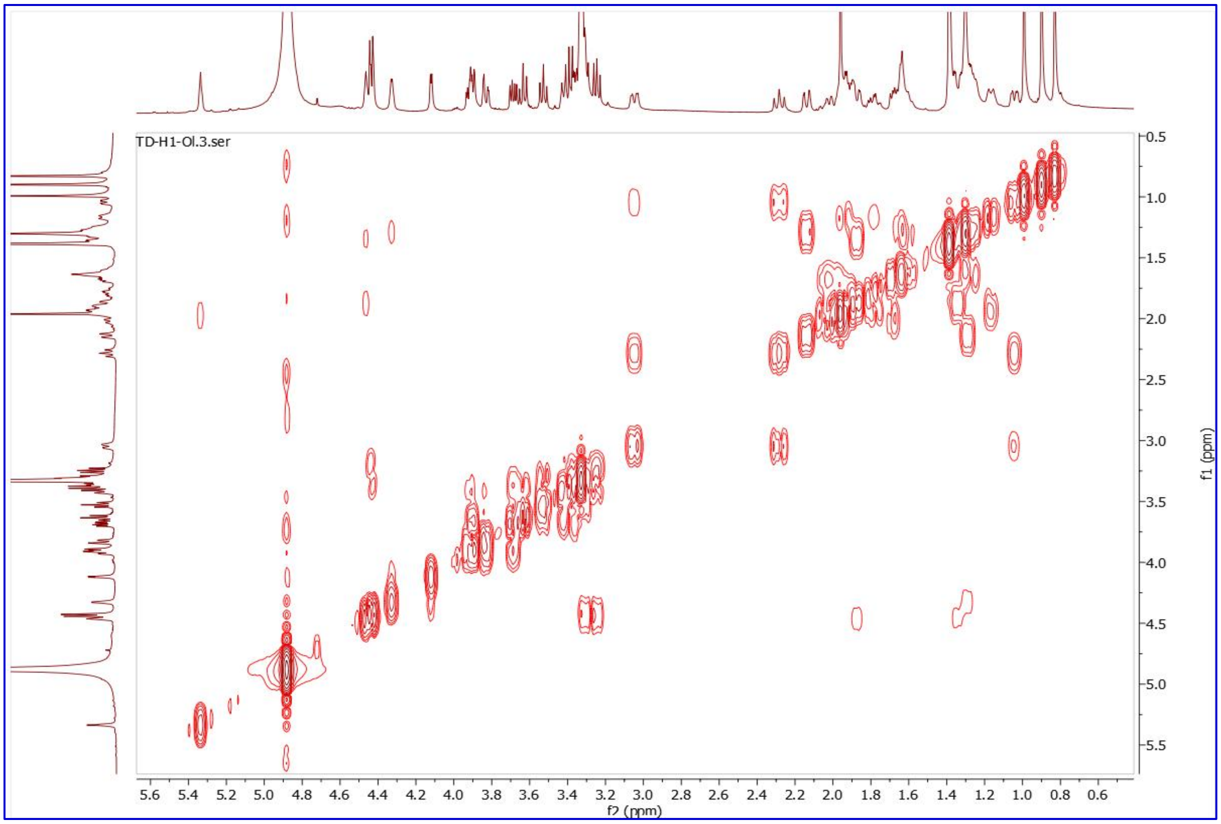

Figure S35. COSY of Prodavaeanoside (**5a**)

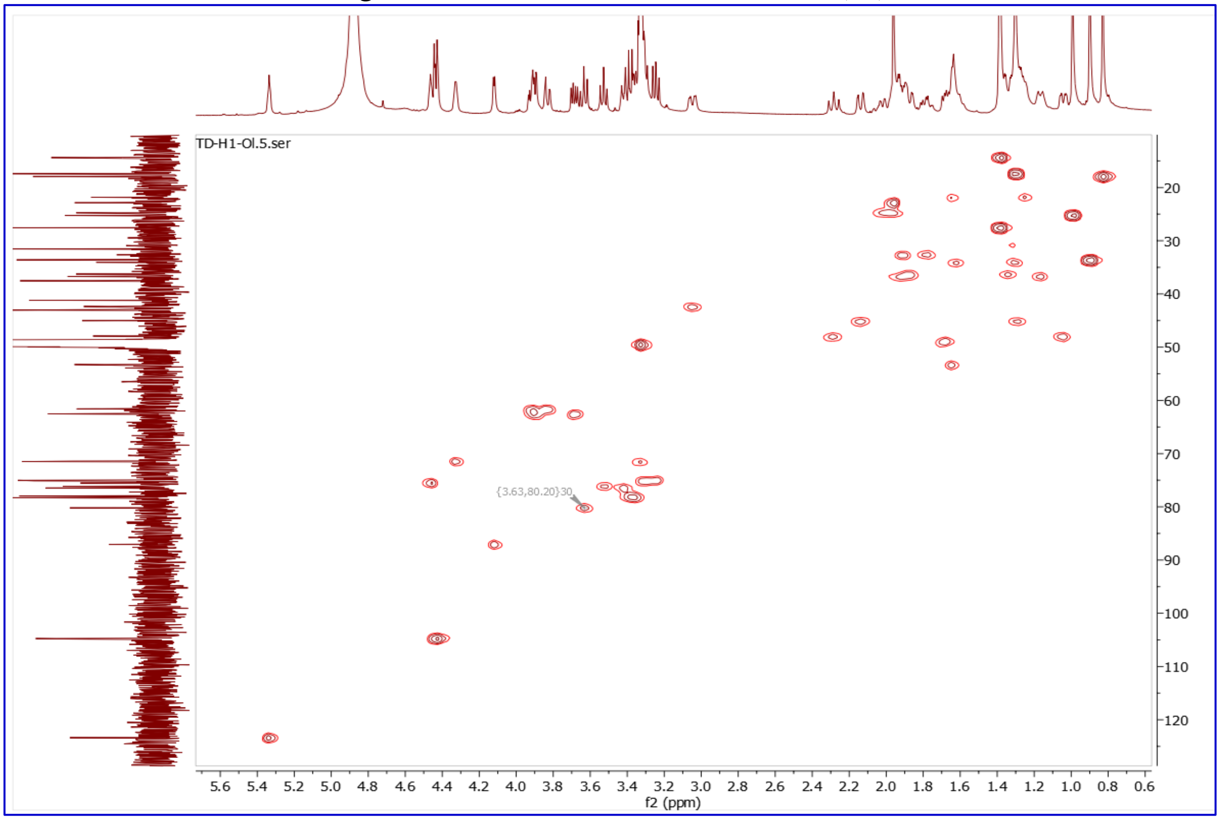

Figure S36. HSQC of Prodavaeanoside (**5a**)

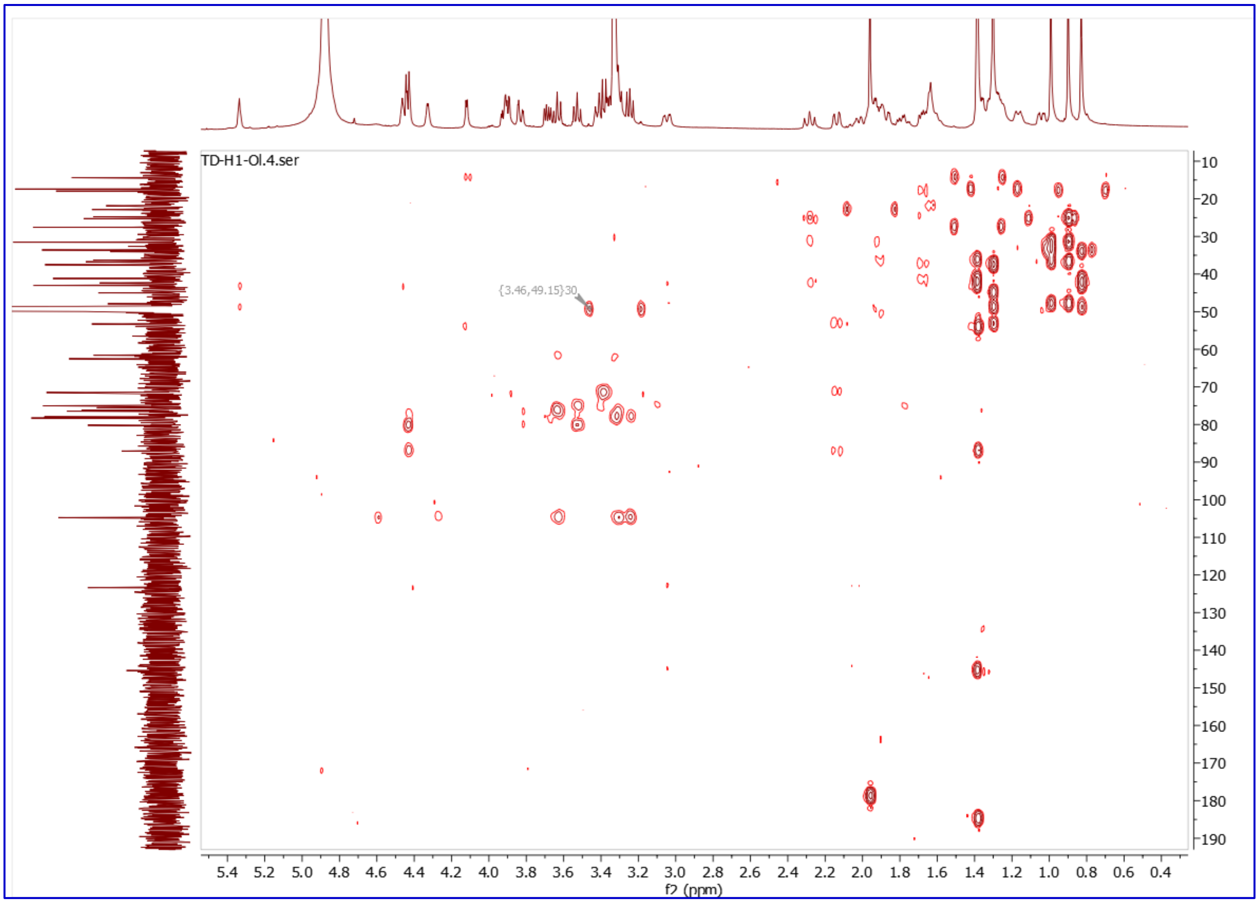

Figure S37. HMBC of Prodavaeanoside (**5a**)

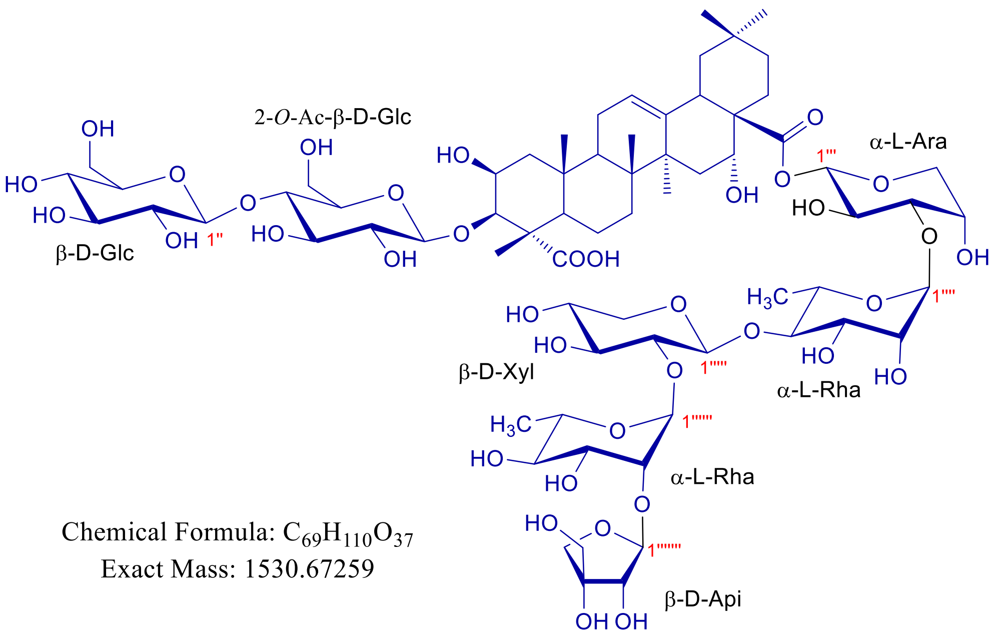

Figure S38. Deacetyl-Daveaenoside (**5b**).

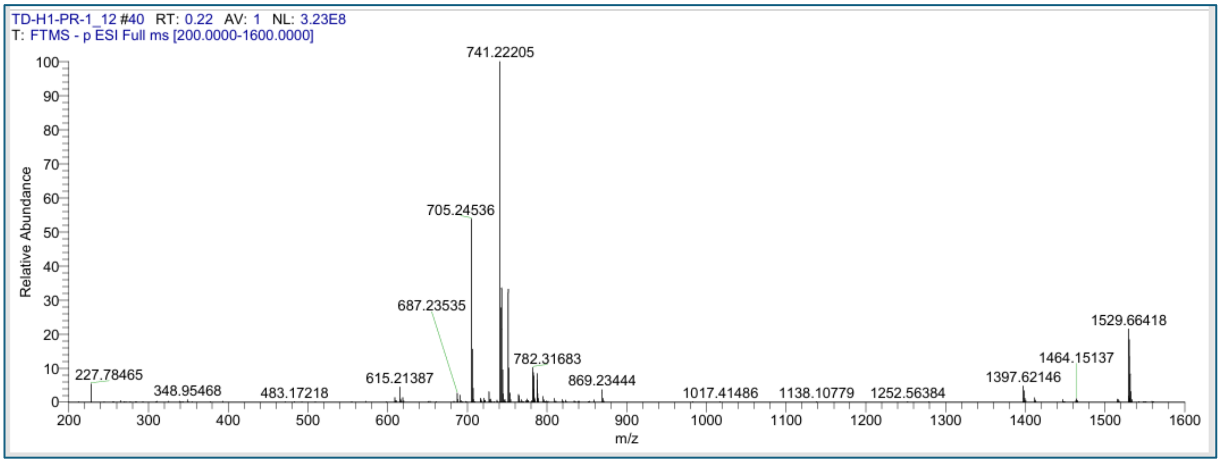

Figure S39. Negative ion ESI-MS of Deacetyl-davaeanoside (**5b**).

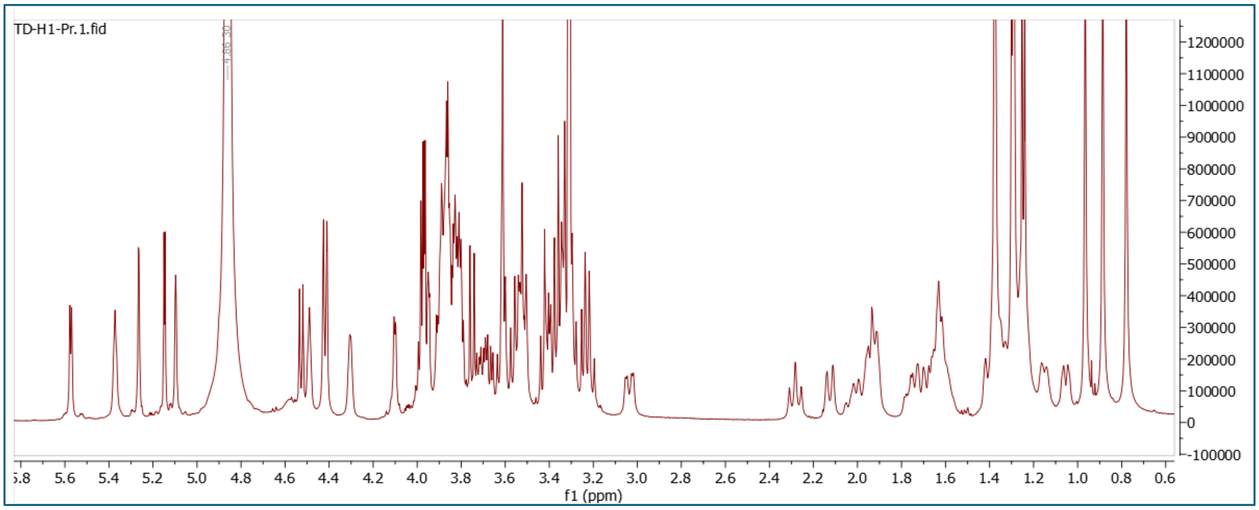

Figure S40. The ^{1}H-NMR Spectrum of Deacetyl-davaeanoside (**5b**) (δ_H 500 MHz, CD_3OD).

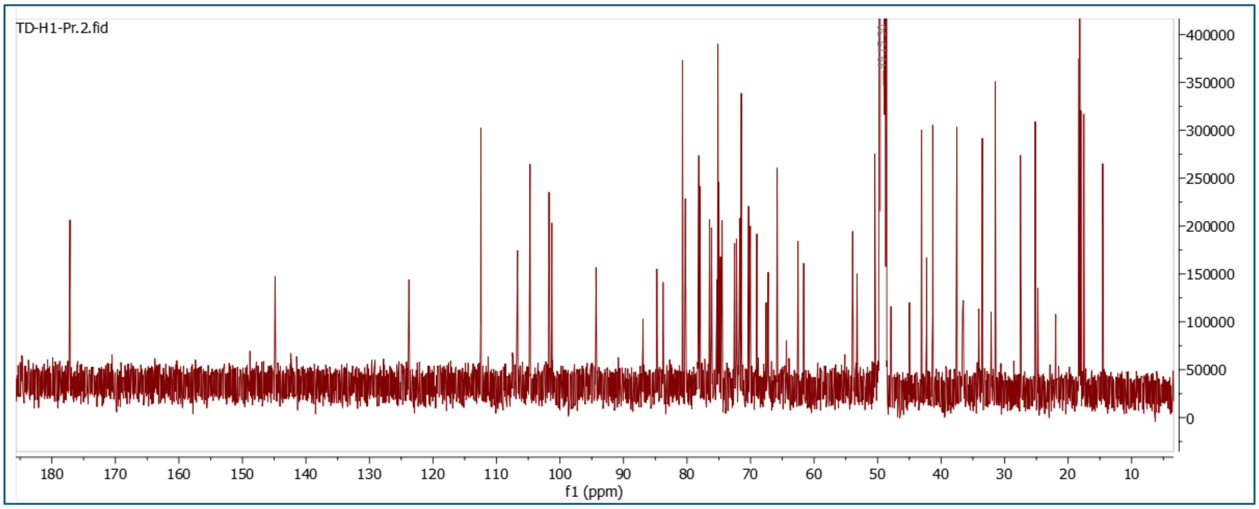

Figure S41. The ^{13}C-NMR Spectrum of Deacetyl-davaeanoside (**5b**) (δ_H 125 MHz, CD_3OD).

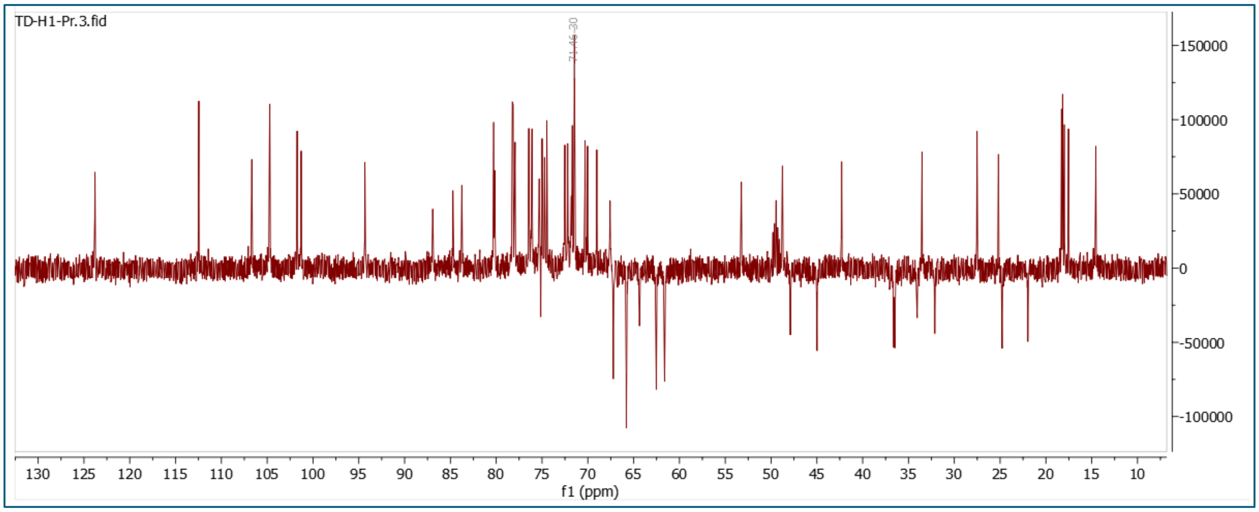

Figure S42. DEPT-135 of Deacetyl-davaeanoside (**5b**)

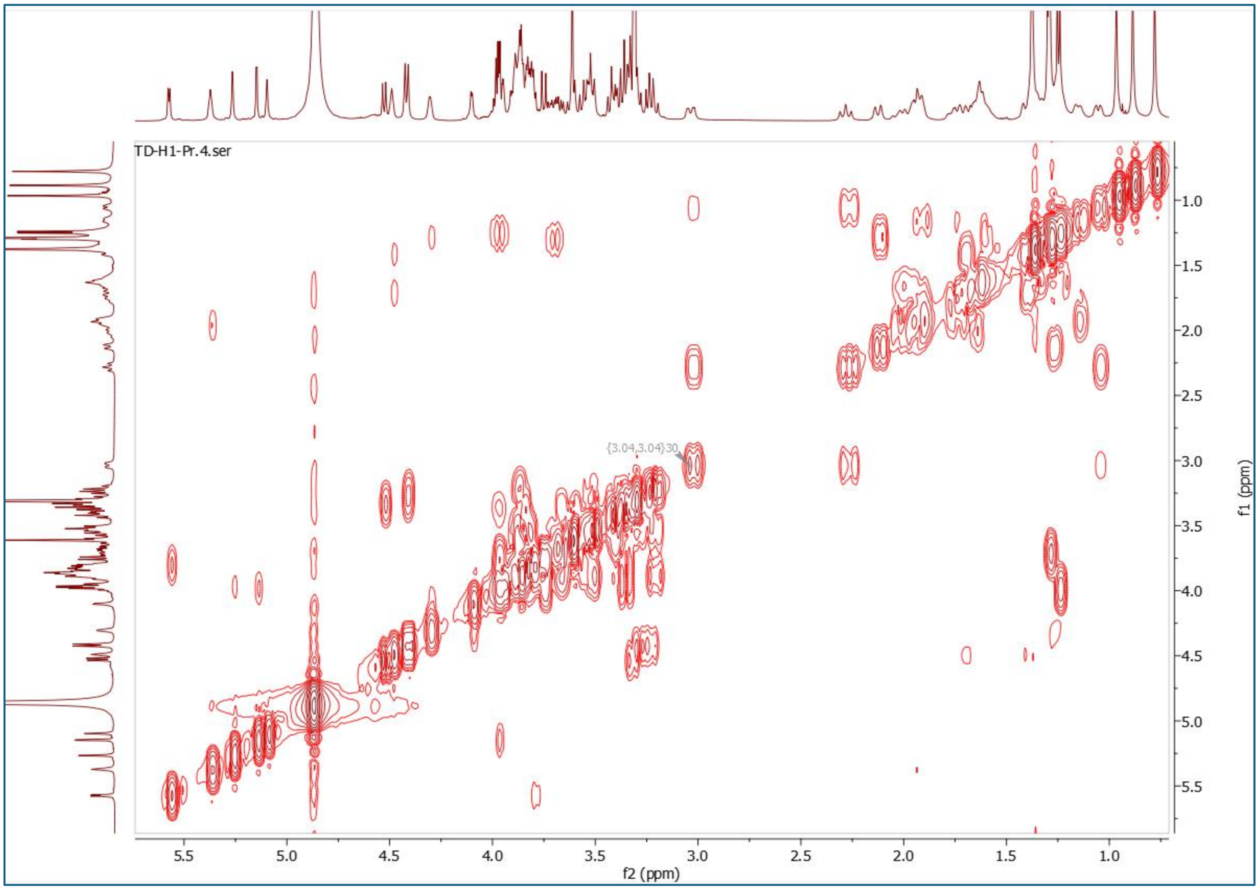

Figure S43. COSY of Deacetyl-davaeanoside (**5b**)

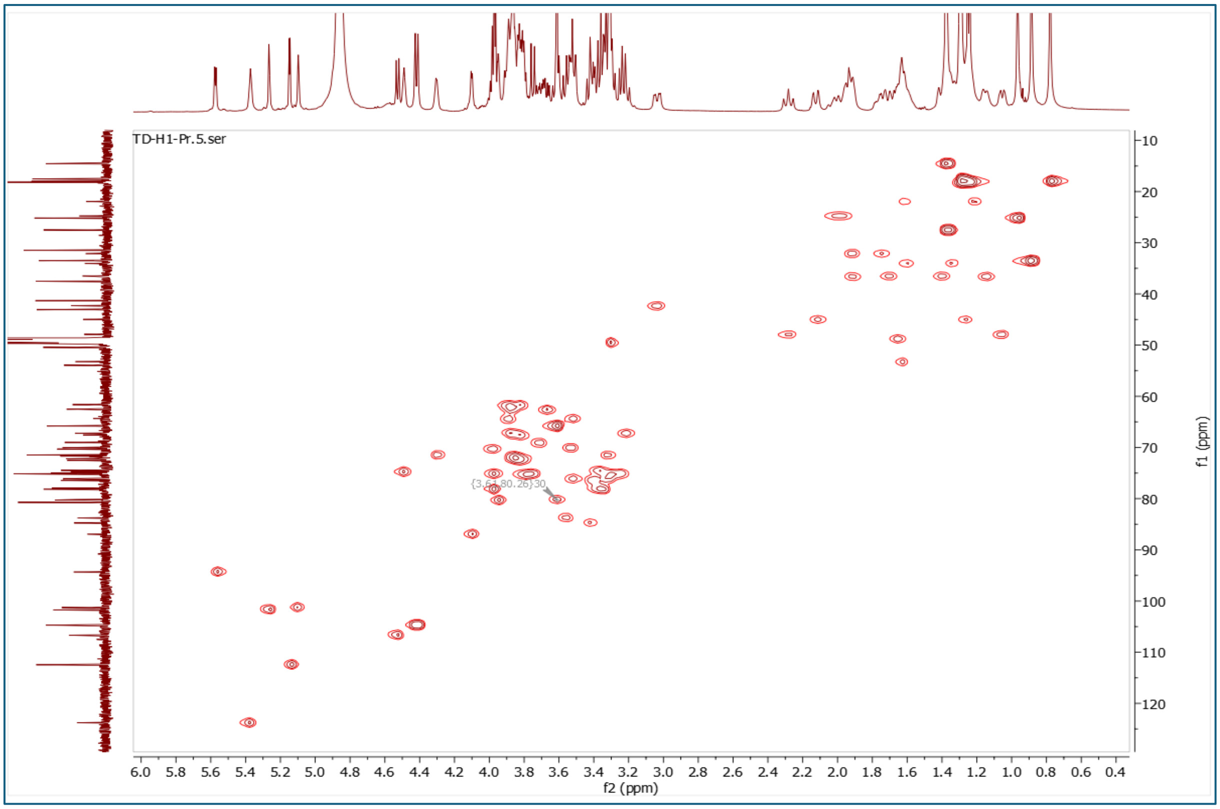

Figure S44. HSQC of Deacetyl-davaeanoside (**5b**)

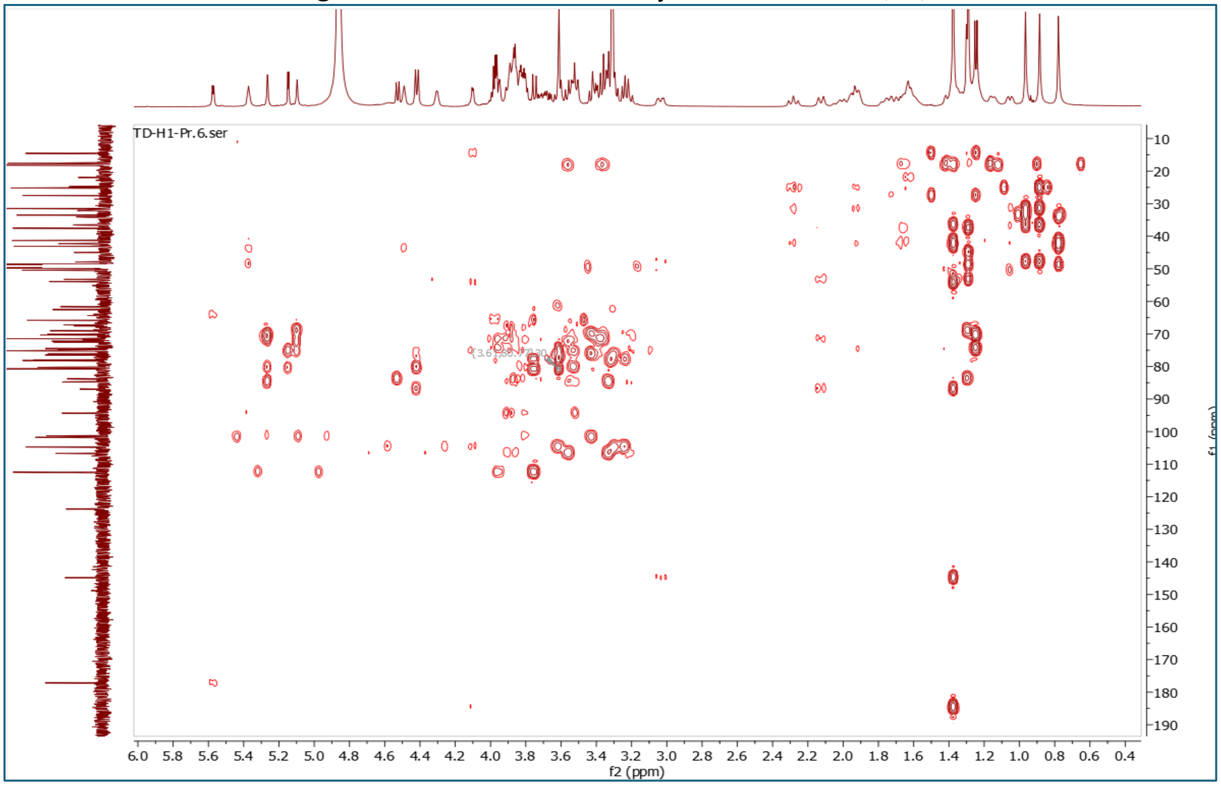

Figure S45. HMBC of Deacetyl-davaeanoside (**5b**)
